# Validation and perception of a key feature problem examination in neurology

**DOI:** 10.1371/journal.pone.0224131

**Published:** 2019-10-18

**Authors:** Meike Grumer, Peter Brüstle, Johann Lambeck, Silke Biller, Jochen Brich

**Affiliations:** 1 Department of Neurology and Neuroscience, Medical Center, University of Freiburg, Freiburg, Germany; 2 Center of Competence for the Evaluation of Teaching in Medicine Baden-Württemberg, Albert-Ludwigs-University Freiburg, Freiburg, Germany; Chinese Academy of Medical Sciences and Peking Union Medical College, CHINA

## Abstract

**Objective:**

To validate a newly-developed Key Feature Problem Examination (KFPE) in neurology, and to examine how it is perceived by students.

**Methods:**

We have developed a formative KFPE containing 12 key feature problems and 44 key feature items. The key feature problems covered four typical clinical situations. The items were presented in short- and long-menu question formats. Third- and fourth-year medical students undergoing the Neurology Course at our department participated in this study. The students' perception of the KFPE was assessed via a questionnaire. Students also had to pass a summative multiple-choice question examination (MCQE) containing 39 Type-A questions. All key feature and multiple-choice questions were classified using a modified Bloom’s taxonomy.

**Results:**

The results from 81 KFPE participants were analyzed. The average score was 6.7/12 points. Cronbach’s alpha for the 12 key-feature problems was 0.53. Item difficulty level scores were between 0.39 and 0.77, and item-total correlations between 0.05 and 0.36. Thirty-two key feature items of the KFPE were categorized as testers of comprehension, application and problem-solving, and 12 questions as testers of knowledge (MCQE: 15 comprehension and 24 knowledge, respectively). Overall correlations between the KFPE and the MCQE were intermediate. The KFPE was perceived well by the students.

**Conclusions:**

Adherence to previously-established principles enables the creation of a valid KFPE in the field of Neurology.

## Introduction

Medical students often find clinical reasoning a particularly difficult topic in the field of neurology. Indeed, the complex structure of the nervous system requires a profound knowledge of neuroanatomy, obtaining a case history can be complicated, especially if patients only provide a vague description of their symptoms, and interpretation of the neurological examination is challenging [1–4]. However, the ability to integrate all this information is necessary for clinical reasoning [5]. Since these skills are not only difficult to acquire, but also to teach, the assessment of clinical reasoning skills serves as an essential form of feedback on these complex processes for students and teachers.

An established approach for assessing clinical reasoning is the "key feature" approach, which was developed in the 1980s by Bordage and Page [6,7]. A key feature (KF) is defined as a critical step in the process of solving of a specific clinical problem. Alternatively, KFs can focus on steps in which examinees are most likely to make errors in the solution of the problem, or can capture difficult aspects of practical problem-identification and management [8]. The KFs are embedded in a key feature problem (KFP), which consists of a clinical case scenario followed by 2–4 KFs. Two types of question formats are applied: (i) the “short-menu” (SM), where examinees have to select their responses from prepared lists, which typically contain 10–30 options that also include common misconceptions to reduce cueing effects [9], and (ii) the “write in” format, which is often replaced by the ‘‘long-menu” (LM) format comprising long lists of possible answers (over 500). Since the LM format is very time-consuming and prone to error when used in a pencil and paper exam [10], computerized assessment tools have been developed to overcome these difficulties [11–13].

To date, there is no KFPE available that has been specifically devised for neurology. The aim of this study was therefore to validate a newly-developed KFPE in the field of neurology, and to examine it is perceived by the students.

## Methods

### General context

The neurology course at the Department of Neurology and Neuroscience, University Medical Center Freiburg, usually takes place during the students’ 3^rd^ or 4^th^ year of study. For the purpose of the present study, the 6-week block course consisted of 12 disease-oriented lectures (max. 80 students) and included a mandatory 3-week block comprising symptom-oriented seminars or Team-based Learning (TBL) units (max. 20 students), practical training for the neurological examination, and bedside-teaching in a small-group setting (6 students). The course finished with a KFPE and multiple-choice question examination (MCQE).

### Key feature problem examination (KFPE)

Using the steps recommended by Page et al. [8], the key feature problems (KFPs) were developed by didactically- and clinically-experienced board-certified neurologists from our Department. The 4 topics of the symptom-oriented seminars/TBL-units (“vertigo”, “acute back pain”, “first epileptic seizure” and “acute altered mental status”) are all part of the German competency-based curriculum (NKLM) [14], and served as domains for the clinical problems sampled in the KFPE. A two-dimensional blueprint based on clinical setting (outpatient vs. emergency room) and frequency (common vs. rare) was adopted for the KFPE, based on students’ detailed notes sourced from the university’s learning management system. Three typical clinical situations for each of the 4 topics were designed, serving as a basis for defining the KFPs. Three or 4 KFs were defined per situation, resulting in a total of 44 KFs. A final case scenario was written in accordance to the KFs, resulting in 12 KFPs. All KFPs were reviewed for their relevance to the contents of the seminars/TBL units and edited for clarity and possible ambiguities; this task was carried out by 2 board-certified neurologists with long-term clinical expertise in neurology and who were not involved as authors of this study. An additional board-certified neurologist with didactic expertise then rechecked the KFs for any other common item flaws. The KFPE was piloted on 26 students who had taken the neurology course 6 months prior to the study. Problems encountered during the pilot phase were addressed before using the KFPs in this study.

Since we used an electronic approach, each KF question could only be answered once, allowing the correct answer to be revealed in the following question item. Backward navigation was possible for reviewing information but not for editing, thus enabling students to avoid subsequent errors. The question formats used were the short-menu (22 KFs, each with 10–20 options) and long menu (also with 22 KFs) [11–13]. The results of all the long-menu answers were double-checked by hand. Partial credits for each correct response were assigned, resulting in a possible maximum score of “1” per KF question. The question scores within the KFPs were averaged so that each KFP had a possible maximum score of “1”, resulting in a possible maximum of 12 points. The KFPE was conducted in the faculty computer lab using a computer-based examination system [15]. Students first viewed a short presentation on the test procedure and the electronic test tool, and then had 60 minutes to complete the KFPE.

### Multiple-choice question examination (MCQE)

The MCQE consisted of 39 questions. All questions were type A multiple choice questions with a set of 5 options, each developed according to guidelines [16] by didactically- and clinically-experienced neurologists from our Department. Questions were developed using a two-dimensional blueprint analogous to the one used for the KFPEs, and were based on the contents of the complete neurology course (the 4 seminar/TBL-unit topics mentioned above, as well as the complementary lecture and bedside-teaching topics such as the neurological examination, stroke, multiple sclerosis, Parkinson’s disease, dementia, myopathies, neurooncology etc.). Three experienced board-certified neurologists internally reviewed all questions. This process resulted in 39 questions, 16 of them referring to the four topics of “vertigo”, “acute back pain”, “first epileptic seizure” and “aAMS”.

### Modified Bloom’s categorization of Key Feature and Multiple-Choice questions

Each question from the KFPE and the MCQE was categorized independently by 3 assessors (physicians with long-time experience in neurology and/or assessment), according to a modified Bloom’s taxonomy of the level of cognitive skill tested (analogous to Palmer et al. [17,18]), into three levels: Level I: Knowledge–recall of information; Level II: Comprehension and application–understanding and being able to interpret data; Level III: Problem-solving–use of knowledge and understanding in new circumstances. The assessors rated the questions independently. Questions that were scored disparagingly were discussed, and all 3 assessors then agreed on a final categorization score for each question.

### Questionnaire for evaluation

The questionnaire (adapted from [13]) consisted of 22 items related to the examinees’ acceptance and appreciation of the KFPE. A Likert scale from 1 (total disagreement) to 5 (total agreement) was used.

### Statistical analysis

We assessed normality distribution with the Shapiro–Wilk test for the distribution of the results of the KFPE and the MCQE. Both showed normality distribution Item analyses (difficulty, item-total correlations) were computed for the KFPE and MCQE using Cronbach’s α to determine internal consistency. Correlations between the KFPE and MCQE were calculated by applying Pearson correlations. Differences between correlations were tested by means of t-tests. Significance levels were set at p < 0.05. All statistical analyses were performed with SPSS software (Version 21).

### Standard protocols, registration and participants’ consent

The study was approved by the local ethics committee, and all participating students provided written informed consent.

## Results

### Performance and reliability of the KFPE

**Fig 1:**
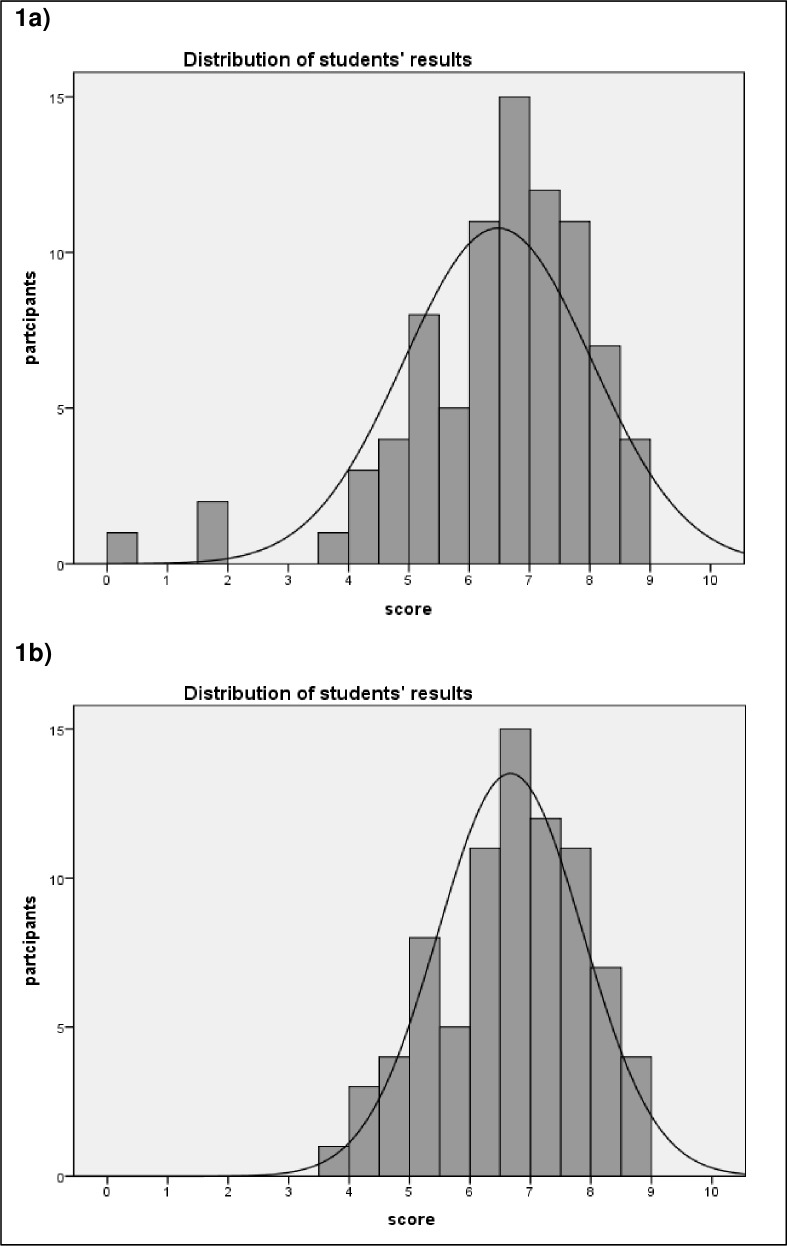
Statistical analysis of the KFPE. 1a) Students' KFPE results, n = 84; average score: 6.47 points, SD = 1.56 1b) Students' KFPE results after exclusion of the 3 outliers, n = 81; average score: 6.67 points, SD = 1.20.

Of the 122 students (92 3^rd^-year and 30 4^th^-year, 70 female and 52 male) undergoing the neurology course, 84 participated in the KFE. They scored an average of 6.5 / 12 points (54.1%, minimum 0.33 points, maximum 8.93 points; SD 1.56). Cronbach’s alpha calculated for the 12 KFP was 0.73 for all 84 participants. The histogram in **Fig 1** illustrates the distribution of the students’ results.

Three participants had markedly lower scores (0.33, 1.66 and 1.78 points). Detailed analyses revealed that one of these three had to quit the KFPE for health reasons after answering only a few questions, the two others quit the KFPE after completion of 2 respectively 3 KFPs without giving reasons. Based on recommendations by Moeltner et al. [19], we excluded these 3 outliers from further analysis due to the vulnerability of both reliability and item-total correlations. After this correction, analysis of the KFE was based on a group of 81 participants (61 3^rd^-year and 20 4^th^-year; 46 female, 35 male). The 81 participants had an average score of 6.7 (55.8%, minimal 4.44 points, maximal 8.93 points; SD 1,2), and Cronbach’s alpha calculated for the 12 KFPs was 0.53 for these 81 participants (**Fig 1**). The item difficulty level for all 12 KFPs was between 0.39 and 0.77. There was no difference in difficulty level between SM- and LM-format questions. (0.55 vs. 0.56 on average). All KFPs had positive item-total correlations, with 7 KFPs reaching the recommended item-total correlation of ≥0.2 (**Table 1**) [19].

**Table 1:** Item difficulty level.

	KFP 1	KFP 2	KFP 3	KFP 4	KFP 5	KFP 6	KFP 7	KFP 8	KFP 9	KFP 10	KFP 11	KFP 12
Item difficulty level	0,64	0,60	0,54	0,55	0,39	0,65	0,77	0,64	0,43	0,62	0,53	0,49
Item total correlation	0,05	0,05	0,30	0,13	0,17	0,29	0,21	0,23	0,36	0,21	0,20	0,15

Item difficulty level: values from 0 to 1. Describes the average point value reached by students in this particular KFP. Item total correlation: values from -1 to +1. KFP: Key Feature Problem

### Results of the MCQE

The MCQE was completed by 122 students. They scored an average of 32.6 (83.6%) out of a possible 39 points. Cronbach’s alpha for the MCQE was 0.76. Eighty-one students took both exams so that their results could be correlated. These 81 students had a mean MCQE score of 32.0 points (82.0%).

### Modified Bloom’s categorization for Key Feature and Multiple-Choice questions

According to a modified Bloom’s taxonomy (see Methods), the KFPE consisted of 12 Level I (27%), 13 Level II (30%), and 19 Level III KF questions (43%). The 3 levels were equally distributed amongst all topics and answer formats (SM and LM).

**Fig 2:**
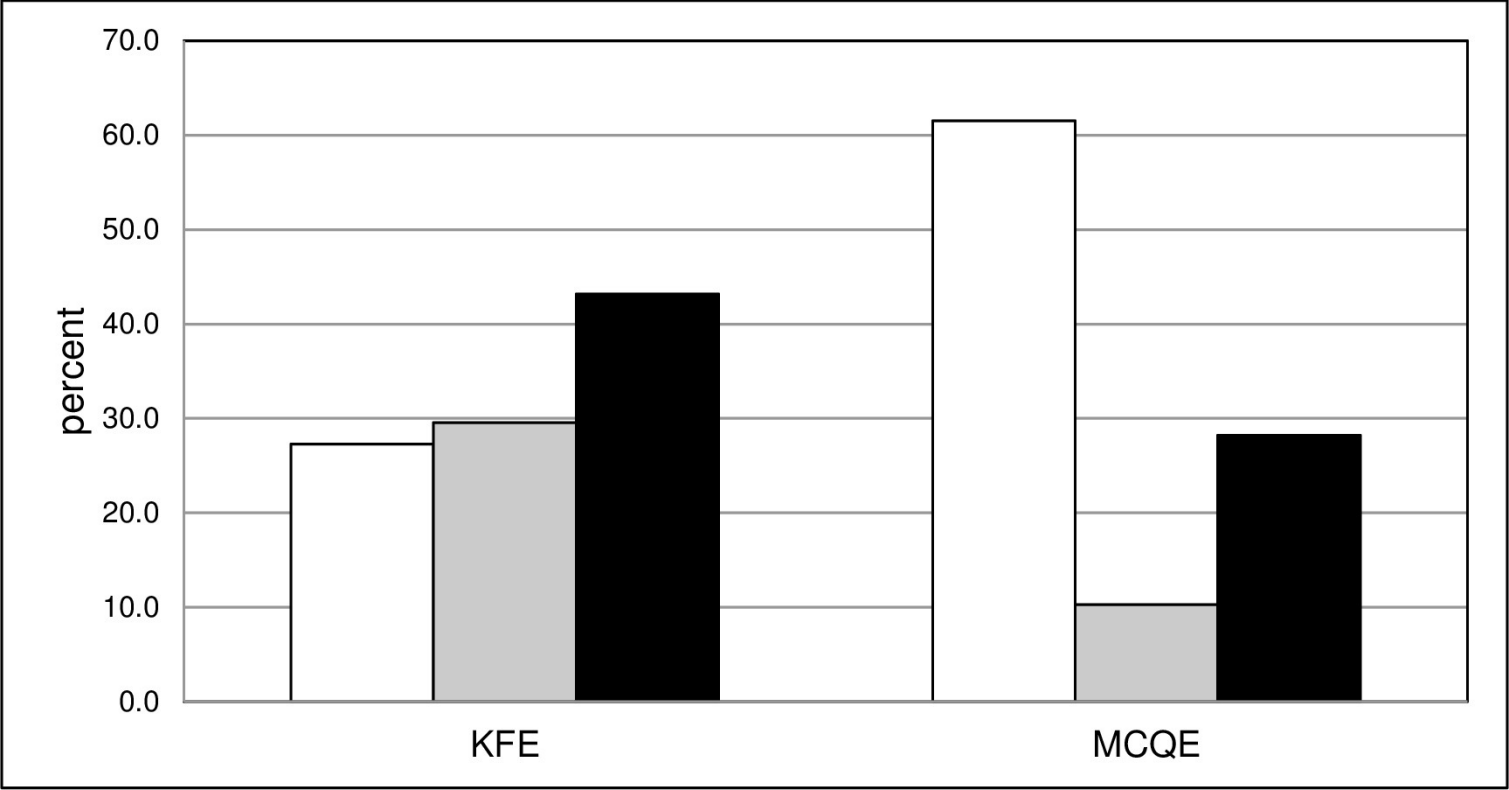
Modified Bloom’s categorization for KFPE and MCQE questions. White column: Modified Bloom’s Level 1; Grey column: Modified Bloom’s Level 2; Black column: Modified Bloom’s Level 3.

In contrast, the MCQE consisted of 24 Level I (62%), 4 Level II (10%), and 11 Level III questions (28%). Of the 16 questions referring to the seminar/TBL-unit topics, 8 were rated as Level I, 1 as Level II and 7 as Level III. The categorization results of all KFPE and MCQE questions are shown in **Fig 2.**

For further analysis, we used an approach analogous to Palmer et al. [17]: Level II and III questions, which both tested for comprehension, application and problem-solving were pooled in order to distinguish them from Level I questions, which tested for knowledge. This resulted in 12 Level I and 32 Level II and III questions for the KFPE, and 24 Level I and 15 Level II and III questions for the MCQE.

In both exams there were no significant differences between the average item difficulty of Level I vs. Level II- and III questions (KFPE: Level I questions: 0.55 vs. Level II und III questions: 0.59; MCQE: Level 1 questions: 0.84 vs. Level II- und III-questions: 0.82).

### Correlations between the KFPE and the MCQE

There was an intermediate correlation between the KFPE and the complete MCQE (0,365; p = 0.001).

### Students’ perception of the KFPE

Eighty-one students completed the questionnaire. Results are shown in **Table 2**.

**Table 2:** Students’ perception of the KFPE.

		Mean	SD
1.	Getting to know this kind of assessment method (case- and computer-based) was interesting to me.	4,44	0,91
2.	Overall, the examination was fun.	3,69	1,03
3.	I felt the level of difficulty was appropriate for the assessment.	3,46	0,94
4.	The key features are problem-oriented.	4,44	0,68
5.	The key features are interdisciplinary.	3,44	0,91
6.	The key features relate closely to the problems that can arise in clinical practice.	4,26	0,59
7.	I enjoyed working on the short cases.	3,74	1,02
8.	Working on the short key features is a useful way of assessing my knowledge.	4,00	1,05
9.	I wish to have an examination with key features in the future curriculum.	3,79	1,13
10.	Being able to select answers from a long list is a good compromise between MC answers and open text answers.	3,82	0,94
11.	My preferred answer was included in the long-menu list.	3,72	0,76
12.	The time frame allowed for the key features was appropriate.	3,72	1,05
13.	The computer-based format of the examination was appealing to me.	3,56	0,99
14.	Working on the key features and questions on the computer was more strenuous compared to pencil and paper examinations.	2,69	1,34
15.	Assessment should preferably be done on computers in the future.	3,10	1,17
16.	The planned time schedule of the examination ran without any problems.	4,64	0,71
17.	The software ran smoothly without technical problems.	4,82	0,45
18.	The screen design was appropriate for conducting a computer-based examination.	4,28	0,83
19.	The text was legible.	4,64	0,58

N = 81; Mean results of a 5-point Likert Scale from 1 (total disagreement) to 5 (total agreement) are shown. SD: standard deviation

## Discussion

After successfully establishing and evaluating a KFPE in the field of neurology, we provide evidence for validity applying a widely-accepted approach based on five sources: Content, response process, internal structure, relation to other variables and consequences [20–22]. In addition, we discuss the topics of item cost-feasibility [23] and approval [24].

### Evidence based on content

Evidence for the validity of our KFPE content was provided by different approaches. We chose as the domains of interest for our KFPE 4 common neurological symptoms, which are all key topics in neurology training in our curriculum. We ensured that the content was representative by applying a two-dimensional blueprint, while its adequacy and relevance to the content of the seminar/TBL units was reviewed by two board-certified neurologists with long-term clinical expertise in neurology. Furthermore, the quality of our questions and their ability to measure challenging decisions was validated by another board-certified neurologist with didactic expertise, who rechecked all KFPs.

### Evidence based on response process

Our participants were familiarized with the KFPE by undergoing a short, standardized introduction, and were supported throughout the test by two supervisors who were familiar with both the test and the computer system. We used short- and long-answer formats, with the long answer response format serving as the electronic equivalent of the write-in format [11]. This format was rated fairly well by our participants in terms of finding the preferred answer, and was deemed to be a good compromise between MCQ answers and free text answers. The scoring system for our KFPE was adapted from Page et al. [7], applying equal weighting for KFs within each case, which were then averaged to generate a KFP score of 1. This approach makes the KFP—rather than the individual KF—the unit of measurement, in light of the item independence assumption in psychometrics [25]. In line with a recommendation by Eva et al. [26,27], we used lay terms to describe the clinical case in order to maximize authenticity and construct-relevant variance. The rating process was supported by an electronic system, but all long-format answers were double-checked manually. This process can be optimized in future applications of the KFPE, since the likelihood of failing to rate the correct answers accurately is rare after several passages. The pilot testing phase of our KFPE was also helpful for this process.

### Evidence based on internal structure

Our KFPE with 12 KFPs initially achieved an reliability score of 0,73 (Cronbach’s alpha calculated for key-feature problems), which was relatively high compared to the results from previous undergraduate examinations (Hatala [28]: 15 KFP: Cronbach’s alpha 0,49; Fischer [13] 15 KFP: Cronbach’s alpha 0,65). Further scrutiny of the students’ results revealed 3 outliers, who achieved markedly-lower results for reasons not associated with the KFPE itself. We excluded these outliers from further analysis, since the item analyses would have otherwise been prone to misinterpretation. This correction resulted in a reduced yet acceptable value for Cronbach’s alpha (0.53), remaining within the range of the above-mentioned results. Furthermore, Cronbach’s alpha of 0.53 for our KFPE is still a remarkable result when compared to that of Hatala et al. [28], since our KFE only consisted of 12 KFPs that had to be answered within 60 minutes for practical reasons. In addition, our KFPE was formative, and participation was triggered mainly by students’ motivation for receiving feedback about their level of clinical reasoning skills. Indeed, this may have resulted in a potentially artificial population of highly-motivated and interested students; under “regular” conditions, the spectrum of student performance is more likely to vary, resulting in a more widespread performance and thereby higher degree of internal consistency. Regarding item difficulty, only acceptable scores between 0.39 and 0.77 were achieved, reflecting the thorough review process. For item-total correlation, all KFPs showed positive correlations. Two of the KFPs (KFP1 and KFP2) resulted in very low levels of item-total correlation; however, the content validation process showed a high relevance of the underlying aspects, so the results of the item analyses are helpful in raising awareness about the different wording of these KFPs.

### Evidence based on relations to other variables

Since our learning objectives for the seminars and TBL units were at the application and clinical reasoning levels, we demonstrated by way of a modified Bloom’s taxonomy classification [17,18] that a clear majority of our KFPE questions implicitly tested for comprehension, application and problem-solving. By closely adhering to instructions for creating key-feature problems [8], our results are in line with those yielded from a similar rating of key feature questions in a KFPE that tested for nutrition [29]. A multi-level review process, along with pilot testing of the KFPE, enabled detection of ambiguities in terms of contents and clarity of phrasing, and this, in turn, ensured that the KF questions were of high quality.

Another source of validity is the relationship between assessment scores and criterion measure scores. We found that there was only a moderate overall correlation between student performance in the KFPE vs. the MCQE. This result is in line with previous studies [13,29,30], and further supports the assumption that different levels of knowledge can be measured, i.e., factual knowledge (“knows”) is measured by the MCQE and clinical reasoning skills (“knows how”) by the KFPE. Think-aloud protocols could serve as a future methodological approach to further elucidate this finding. However, until then, these results argue against simply using higher-level MCQ as a substitute for key feature questions for assessing clinical reasoning, even though it is still possible to achieve the levels of comprehension, application and problem-solving in MC questions with modified Bloom’s taxonomy.

### Evidence based on the consequences of testing

The consequential aspect of validity refers to how the KFPE impacts on teaching and learning aspects as well as its influence on the faculty. In this context, it is interesting to note that student performance in the KFPE was markedly lower than that in the MCQE, which was also observed in previous studies [13, 28, 31]. These results point to the assumption that, in contrast to their high level of factual knowledge, the participating students' reasoning skills are open to improvement; this, for example, could be achieved through optimized and adequate teaching methods. Teaching formats that foster clinical reasoning skills such as problem-based [32] or team-based [33,34] learning may help students to develop more expertise in this important field.

### Evidence based on acceptability and cost-feasibility

In addition to the above-mentioned sources of validity, Van der Vleuten established 2 other criteria for determining the utility of assessment methods [35]: acceptability and cost of the assessment method. The results of the questionnaire demonstrated that the participants showed a high level of acceptance of and appreciation for the KFPE. They especially rated the KFPE as a useful way of providing feedback on their clinical decision abilities. Furthermore, the questions that evaluated the electronic implementation of the examination even yielded slightly better results than those of Fischer et al. from almost 10 years ago [13], pointing to a greater familiarity in dealing with electronic formats. This is also pertains to costs of running the KFPE: Once the computer-based examination system is established, the process of conducting and analyzing the test will be inexpensive, since most faculties already have a computer pool, and upcoming tablet-based solutions are generating more flexibility by enabling electronic examinations to be carried out in a lecture hall.

There are several limitations to this study. 3rd and 4th year medical students were both included in the analysis. Also, it seems generally plausible, that 4th year medical students appear to have more experience in clinical reasoning compared to 3rd year medical students, this does not apply to the students at our University of Freiburg: We have a stringent curriculum, scheduling Neurology in only one position. That implicates, that all students—independently from their year of clinical studies—have the first contact with Neurology in our course, making their clinical reasoning skills in Neurology well comparably. In addition, reasons like interruption time for the initiation of the medical thesis, studies abroad with a different curriculum, parental leave, etc., lead to deferrals in study years without having more clinical experience. Only 84 of 122 students participated in KFPE, causing a possible selection bias. The key feature problem examination (KFPE) was a mandatory formative examination for all students, although an authorized absence was allowed if none of the other teaching units had been missed. Since the KFPE took place at the very end of the course, we could not evaluate the reasons for nonattendance. We can only speculate that KFPE participants were more interested in receiving feedback on their clinical decision reasoning skills than the nonparticipants. The equal results of participants vs. nonparticipants in the MCQE (data not shown) indicate no difference in the academic level. Despite this attenuated number of participants, this sample size comprises the largest set of students in the world undertaking a complete KFPE in neurology.

The results represent a single-center experience, and although the KFPE passed through a multi-level review process, item analysis revealed that several KFPs barely met the target values for item-total-correlation, thus decreasing the internal consistency. The results of the item analyses now serve as a valuable basis required for further improvement and development of KFPs. The KFPE was formative, resulting in a possible selection bias, with a consecutively-restricted population of participants (see above). Internal consistency was even lower after exclusion of the 3 lowest-scoring participants. Internal consistency could therefore be improved by applying the KFPE as a summative examination to all neurology students, as well as adding more key-feature problems in the test, which, on the other hand, could interfere with the feasibility of the KFPE. Although KFPEs represent a suitable tool for assessing students’ clinical reasoning skills during high-stake examinations, and indirect evidence for a correlation with clinical performance does exist [36–38], it still remains unclear whether an increase in KFPE scores is related to a direct increase in clinical performance. Studies addressing this question using performance measures such as the mini-clinical examination exercise (mini-CEX) [39] are thus required.

Taken together, by applying several sources of validity evidence our study demonstrates that it is possible to create a valid and well-received formative KFPE as a tool for assessing clinical reasoning in neurology. The feedback received through the KFPE may not only guide students to 'fill in the gaps' in important and common clinical situations, but can also assist teachers in reviewing the methods that yield the best evidence-teaching of clinical reasoning. Moreover, since it was demonstrated that the KFPE may also be a valid tool for assessing medical residents [31], it may help in structuring their training and providing essential feedback for continued improvement in performance. We therefore encourage other teachers to add this type of examination to the spectrum of their assessment methods.

## Supporting information

S1 File The KFPE in German. (PDF)

S2 File The KFPE in English. (PDF)
