# Supplementary material for: Validation and perception of a key feature problem examination in neurology
Source: PLoS One. 2019 Oct 18;14(10):e0224131. doi: 10.1371/journal.pone.0224131 (PMC6799971; doi:10.1371/journal.pone.0224131)
Supplement: S1 File — (PDF) [file pone.0224131.s001.pdf]

Key Feature Problem Examination (German version)

1 Mr Heinrich is a 74-year-old patient in slightly-reduced general health and with a good nutritional status (1.78 m, 78 kg). He presents to the emergency room (ER) because of a headache that has lasted for 3-4 weeks, and increasing somnolence. He is accompanied by his wife, who reports that her husband is usually a lively and active person. She now reports that he has apathy quite frequently, falls asleep "just like that" and has had a “complete change of personality”. Previous illnesses include a TIA (transient ischemic attack) in 2008, chronic atrial fibrillation, and arterial hypertension. Mr Heinrich reports complete abstinence from alcohol for 10 years and from nicotine for 20 years. His medication consists of the antihypertensive drug ramipril (2,5 mg b.i.d), and phenprocoumon (Marcumar®) for anticoagulation [target INR (international normalized ratio) 2.0-3.0]. His medical report is inconspicuous apart from the known atrial fibrillation, there is no evidence for infection, and Mr Heinrich is afebrile.
The neurological exam reveals Mr Heinrich to be somnolent, but rapidly contactable. He seems generally slowed and cannot concentrate well. Apart from a subjective heaviness that is reported in the pronator drift test (left arm), and slight clumsiness of the left hand, there are no motor or sensory deficits, the cranial nerve exam is normal, and there is no nuchal rigidity. In the “tightrope walker” test, the patient had a tendency to drift to the left, and the Romberg test also revealed a tendency to tip to the left.
The emergency lab tests show an INR of 2.84. All other parameters (blood count, electrolytes, inflammation, hepatic, renal and thyroid parameters) are normal.

1.a What is your tentative diagnosis?
(Short Menu, 1 Answer)
(A) Transient global amnesia (TGA)
(B) Pharmacogenic cause (ramipril and phenprocoumon)
(C) First diagnosis of epilepsy
(D) Chronic alcohol abuse despite the patient claiming otherwise
(E) First diagnosis of dementia
(F) Intracranial mass
(G) Ischemic stroke
(H) Encephalitis
(I) "Parainfectious" encephalopathy

1.b You arrange a brain imaging scan to rule out an intracranial mass. In the CT you see a sickle-shaped mass (with iso-, hyper-, and hypodense parts) that is concave to the brain. It is located between the cortex and skullcap, and extends to double the thickness of the skullcap, with a midline shift of 7 mm.
Which of the following diagnoses would you make now?
(Short Menu, 1 Answer)
(A) Acute subarachnoid bleeding
(B) Acute epidural hematoma
(C) Acute subdural hematoma
(D) Acute meningitis
(E) Acute encephalitis
(F) Internal bleeding from a parietofrontal meningeoma
(G) Glioblastoma

(H) Medulloblastoma
(I) Chronic subarachnoid bleeding
(J) Chronic epidural hematoma
(K) Chronic subdural hematoma
(L) Chronic meningitis
(M) Chronic encephalitis
(N) Leptomeningeal carcinomatosis

1.c You diagnose the patient with a chronic subdural hematoma, with the onset of focal neurological deficits that are associated with a marked space-occupying effect.
What would be the next therapeutic step?
(Long Menu, 1 answer from the following list of accepted options)
Burr hole surgery, Evacuation of the chronically-bleeding hematoma (e.g. through drainage), Surgery, Surgery: evacuation of the subdural hematoma, Subdural hematoma evacuation

2 Mr Rudolf is a slightly-obese 58-year-old patient (1.78 m, 90 kg) in good general health. He presents to the ER with strong back pain (onset 3 hours earlier), and new onset of gait disturbance ("tripping"). He reports frequent back aches, but the current one is "especially severe". He cannot recall any particular triggering situation or trauma. He works as a builder, is married, and has two adult children. He is a non-smoker. Previous illnesses comprise arterial hypertension, an operation for inguinal hernia and a tonsillectomy.
The clinical exam reveals bilateral dorsiflexion palsy and plantar flexion palsy of the foot [Medical Research Council (MRC) scale grade 4], which is more pronounced on the right than the left side. The straight leg raise test (Lasègue`s sign) is positive bilaterally, and sensation is reduced on the right side of the chin, the entire dorsal aspect of the right leg and on both buttocks.
The Achilles tendon reflex is absent bilaterally, and all other tendon reflexes are normal. The remaining basic neurological exam (cranial nerves, nuchal rigidity) is inconspicuous. When asked about bladder and sphincter function, Mr Rudolf reports he has had no urge to go to the toilet in the past 3 hours.

2.a Which additional clinical tests do you now carry out? (Long Menu, 2 answers from the following list of accepted options)
Anal reflex, Cremaster reflex, Bulbocavernous Reflex, Sphincter tone, Anal sphincter tonus

2.b In addition to the abovementioned results of the physical exam, you find reduced anal sphincter tonus and a reduced anal reflex on the right side.
What is your suspected diagnosis now? (Long Menu, 1 answer from the following list of accepted options)
Cauda equina -Cauda syndrome

2.c What would be the next appropriate diagnostic step (additional diagnostic tests)? (Long Menu, 1 answer from the following list of accepted options)
Lumbar MRI, Lumbar magnetic resonance imaging, Magnetic resonance imaging of the lumbar spine, MRI of the lumbar spine, CT of the lumbar spine, Lumbar CT, Lumbar computer tomography, Computer tomography of the lumbar spine

2.d Lumbar MRI reveals a large medial vertebral disc prolapse, with compression of the cauda fibres.

What is the next therapeutic step? (Short Menu, 1 Answer)
(A) Pain relief with diclofenac p.o.
(B) Suggest going to a rehab clinic, since rehabilitation is necessary
(C) Refer the patient to an orthopaedic specialist
(D) Refer the patient to an independent neurologist for follow-up
(E) Watch and wait
(F) Renewed examination of the patient in 2 weeks
(G) Local pain relief with lidocaine s.c.
(H) Emergency surgery
(I) Pain relief with Diclofenac i.m.
(J) Prescription for physiotherapy
(K) Refer the patient to an oncologist for further tests
(L) Local Fentanyl patch
(M) Referral to an independent neurosurgeon for surgical planning
(N) Plan to operate in 3-4 days

3 Mrs. Hackman is a slightly obese 62-year-old patient (1.62 m, 81 kg) in good general health. She presents at your practice with strong rotational vertigo that has been present for 2 days and is especially evident when getting out of bed or after fast movements. She reports fast symptom relief if she immediately lies down and closes her eyes. In addition, she reports strong nausea during the attacks. No further symptoms are reported by Mrs. Hackmann for the intervals between the attacks. Her previous medical history comprises arterial hypertension and hypothyroidism, as well as two operations (appendectomy and hysterectomy).

3.a Which of the following 2 questions are most helpful for further diagnosis of the vertigo? (Short Menu, 2 Answers)
(A) Provoked by movement?
(B) Duration of episode?
(C) Night sweats?
(D) Diarrhoea?
(E) Headache?
(F) Fever?
(G) Weight loss??
(H) Family history?
(I) Previous psychiatric disease?
(J) Improves with sport?
(K) Allergies?
(L) Sensitivity to light?
(M) Cognitive impairment?
(N) Social history?
(O) Pain?
(P) Time spent abroad?

3.b Detailed questioning reveals that the rotational vertigo appears particularly when rotating the head to the right side, and fades away completely after 1-2 minutes in the supine position. You hence suspect benign paroxysmal positional vertigo (BPPV). You carry out a basic neurological exam (nuchal rigidity, cranial nerves, tendon reflexes, sensation, motor function, coordination).

Which of the following results is incompatible with your suspected diagnosis? (Short Menu, 1 answer)
(A) Slight rigidity in the right arm
(B) Bilateral eudiadochokinesis
(C) Negative Romberg test
(D) Negative Unterberger stepping test
(E) Finger-to-nose test secure on both sides
(F) Vibration sense 5/8 in the base joints of both big toes
(G) Exhaustible gaze-evoked nystagmus
(H) Biceps-tendon reflex slightly weaker on the left side
(I) Inconspicuous facial nerve innervation on both sides
(J) Slightly vague temporal orientation (the 24th instead of 26th November)
(K) Head impulse testing positive to the right
(L) Reduced sensation on the inner surface of the left hand
(M) MRC scale grade of 4-5/5 for the left foot flexor
(N) No abdominal skin reflex on either side
(O) Normal hypoglossal nerve innervation on both sides
(P) Head impulse testing negative
(Q) Increased 360 degree turn test (10 steps)

3.c When asked, Mrs Hackmann reports an episode of severe vertigo (duration several days) with associated nausea and vomiting a couple of months ago. Since this resolved by itself after a couple of days, she didn't feel the need to see a doctor.
What is your next diagnostic step regarding the current vertigo attacks? (Long Menu, 1 answer from the following list of accepted options)
Positioning, Dix-Hallpike test, Positioning maneuver, Positioning test, Hallpike test

3.d Your suspected diagnosis of a benign paroxysmal positional vertigo is confirmed. The positioning maneuver towards the right side reveals a rotational nystagmus to the lower ear, with associated rotational vertigo (duration < 1 minute). You successfully carry out the therapeutic positioning maneuver (Sémont) and Mrs Hackmann reports complete remission of her symptoms at her follow-up appointment a week later.
Four months later she presents spontaneously at your practice again with the same vertigo attacks of 1-2 minutes duration.
What is your most likely diagnosis now?
(Long Menu, 1 answer from the following list of accepted options)
Benign paroxysmal positional vertigo, BPPV, Recurrent benign paroxysmal positional vertigo, Recurrent BPPV

4 Mr. Maier is a 58-year-old teacher (1.80 m, 98 kg) who presents to you at the ER. Three hours earlier, passers-by had seen him lying on the ground “twitching” and hence made an emergency call. When the paramedics arrived, they found normal vital signs (blood pressure 128/78 mmHg, heart rate 88/min), and blood glucose was 134 mg/dl. Mr Maier is now fully awake and oriented again, but is still markedly slowed. He remembers neither the “twitching” episode, nor the fall. He reports a general feeling of malaise and has had a cold in the past couple of days; he also reports vomiting once. Mr. Maier states that he has suffered from a strong headache for several days. His medical history includes type-2-diabetes (under dietetic control). You carry out a neurological exam.

4.a Which of the following findings merits particular attention? (Short Menu, 1 Answer)
(A) Pallaesthesia score of 5/8 for the base joint of the big toe on both sides
(B) Slow and erroneous serial subtraction (serial seven), errors when 'RADIO' is spelled backwards
(C) Achilles tendon reflex weakened on both sides
(D) Trömner reflex negative on both sides
(E) Negative palmomental reflex
(F) Exhaustible Glabellar reflex
(G) Exhaustible end-point nystagmus on both sides
(H) Slightly reduced hearing ability on the right
(I) No abdominal skin reflex on both sides

4.b Due to the pathological finding from the bedside neuropsychological exam, you suspect Mr. Maier is suffering from a concentration and attention deficit. Further results of the neurological exam (nuchal rigidity, cranial nerves, motor function, reflexes, sensation) are inconspicuous. The current vital parameters assessed in the ER are now: heart rate 92/min, blood pressure 141/82 mmHg, temperature 38.8°C, blood glucose 121 mg/dl.
What is your suspected diagnosis now? (Long Menu, 1 answer from the following list of accepted options)
Encephalitis

4.c You suspect encephalitis. Concurrently the lab parameters show a white blood count of 13.000/µl (normal: 3.800-10.500/µl) and C-reactive protein (CRP) of 48 mg/l (normal: < 10 mg/l). You hence draw blood cultures. How do you proceed diagnostically?
Name your next 2 diagnostic steps. (Long Menu, 2 answers from the following list of accepted options)
Lumbar puncture, LP, cranial magnetic resonance imaging, cranial MRI, cerebral imaging, cerebral imaging with contrast medium, cCT with contrast medium, cranial CT with contrast medium, PCR anaylsis of the CSF, Polymerase chain reaction analysis of the CSF

4.d CSF analysis reveals pleocytosis (189/µl; normal: < 4/µl) and a total protein concentration of 69 mg/100 ml (normal: < 40 mg/100 ml). Cranial MRI with diffusion-weighted imaging (DWI) reveals a hyperintense lesion in the left temporal lobe.
What is the most likely pathogen? (Long Menu, 1 answer from the following list of accepted options)
Herpes simplex Type 1

5 Mr Hofrichter is a slim 46-year-old man (1.82 m, 84 kg) in good general health. He presents to your practice with slight rotational vertigo and nausea (duration 1 day), and a one-off vomiting episode. He reports difficulty in carrying out his profession (computer scientist), because he cannot operate the computer mouse properly any more. He has also noticed that his gait is unsteady. He is not on any medication and there are no previous diseases to report. He states that he had an operation following a Weber B ankle fracture. Your technician has already performed an ECG, which shows normofrequent sinus rhythm.

5.a What is the next question you ask the patient? Mark the most important question (Short Menu, 1 Answer)
(A) Accompanying tinnitus?
(B) Epigastric aura?

(C) Temporal course of symptom occurrence?
(D) Previous psychiatric diseases?
(E) Panic attacks?
(F) Sensitivity to light?
(G) Provoked by movement?
(H) Situation-dependent improvement/decline?
(I) Improvement through sport?
(J) Weight loss?
(K) Allergies?
(L) Stress at work?
(M) Polyuria and polydyspia?

5.b Mr Hofrichter reports an acute onset ("within 1 minute“) of the vertigo at noon, and said that this has remained roughly unchanged since. He denies having a positional dependency but any changes in position increase his nausea. After you have spoken to him for longer, you think you can detect slight dysarthria, although you are not entirely sure because the patient speaks in heavy dialect. Furthermore, you detect a slight upbeat-nystagmus.
Which of the following exams are particularly important in this case to lead you onto the correct diagnostic pathway? Mark 7 answers:
(Short Menu, 7 Answers)
(A) Gait examination
(B) Pointing tests (finger-to-nose, heel-knee)
(C) Test for meningitis
(D) Diagnostic positioning test
(E) Check for Lhermitte's sign
(F) Test for diadochokinesis
(G) Head-impulse testing
(H) Vibration sensitivity test
(I) Check for Romberg's sign
(J) Test for skew deviation
(K) Examine proprioceptive reflexes
(L) Examine polysynaptic reflexes
(M) Examine tongue movability
(N) Check for epicritical sensitivity
(O) Test for muscle
(P) Check gag reflex
(Q) Check corneal reflex
(R) Examine proprioception
(S) Examine pain sensitivity
(T) Check for nystagmus with Frenzel glasses

5.c Your neurological exam reveals reduced balance and a tendency to fall to the right (even with open eyes during Romberg‘s test), and right-sided hemiataxia. Upbeat nystagmus is confirmed with Frenzel glasses, and the head impulse test is bilaterally negative.
What is your next instrument-based diagnostic test? (Long Menu, 1 answer from the following list of accepted options)
Cranial magnetic resonance imaging, Cranial MRI, cMRI, Cerebral imaging, Cerebral imaging, CCT without contrast medium, Cranial CT without contrast medium

5.d Cranial MRI reveals a right-sided cerebellar infarction that occupies roughly 2/3 of the cerebellar hemisphere. The infarction is due to a dissection-related occlusion of the right vertebral artery. You start secondary prophylaxis with aspirin 100 mg.
The next morning, Mr Hofrichter initially shows increasing agitation and then progressive somnolence. The vital parameters and the lab parameters are normal.
Which severe neurological complication do you now have to take into account in patients with this condition? (Long Menu, 1 answer from the following list of accepted options)
Brain edema, Brainstem compression, Hydrocephalus occlusus, Herniation, Infratentorial herniation,
Secondary bleeding, CSF blockage

6 Mr Winkelmann is a 45-year-old patient in good general health with a normal diet (1.82m; 80kg). He arrives at the ER with strong back pain (since yesterday) that radiates into the right leg. He reports that sudden onset of pain occurred when he lifted a box of water bottles. He does not have any previous illnesses and is not on any medication; previous operations include an appendectomy. The basic neurological exam (nuchal rigidity, cranial nerves, tendon reflexes, motor function, reflexes, sensation, coordination) reveals a slight loss of sensation in the right anterior lower leg and a right foot dorsiflexion palsy (MRC scale grade 4), while the remaining findings are normal.

6.a Which additional diagnostic steps would help you to distinguish radiculopathy from neuropathy in this case? (Long Menu, 2 answers from the following list of accepted options)
Trendelenburg's sign, Straight leg raise test (Lasègue's sign)

6.b The straight leg raise test and Trendelenburg’s sign are positive on the right side. Where do you suspect the lesion to be? (Short Menu, 1 Answer)
(A) Right nerve root of L5
(B) Right peroneal nerve
(C) Left peroneal nerve
(D) Right tibial nerve
(E) Left tibial nerve
(F) Corticospinal (pyramidal) tract
(G) Internal capsule
(H) Left nerve root of L5
(I) Right nerve root of L4
(J) Left nerve root of L4
(K) Motor endplate
(L) Anterior tibial muscle
(M) Extensor hallucis longus muscle
(N) Gastrocnemius muscle
(O) Right thoracic spinal cord

6.c You suspect right-sided L5-radiculopathy. Which further diagnostic test is mandatory now? (Short Menu, 1 Answer)
(A) Lumbar puncture
(B) MRI (magnetic resonance imaging) of the lumbar spine
(C) CT (computer tomography) of the lumbar spine
(D) EMG (electromyography)
(E) NCS (nerve conduction studies)

(F) No further tests necessary
(G) X-ray of the lumbar spine
(H) Myelography
(I) PET (positron-emission-tomography)
(J) Functional X-ray images of the lumbar spine in flexion and extension
(K) Osteodensitometry
(L) Cerebrospinal fluid (CSF) scintigraphy
(M) SSEP (somatosensory evoked potentials)
(N) Biopsy

6.d You prescribe Diclofenac 75mg bid with concomitant gastric protection. Two days later, the patient presents again with marked amelioration of symptoms.
What is now the best way to proceed? (Short Menu, 3 Answers)
(A) Referral to an orthopedic surgeon
(B) Recommend an immediate follow-up visit in case pareses worsen or bladder/sphincter dysfunction occurs
(C) Operate as soon as possible
(D) Referral for elective surgery
(E) Continue diclofenac p.o. for 1-2 weeks
(F) Refer to a neurosurgeon
(G) Hospital admission
(H) Strict stepped-bed positioning for 2 weeks
(I) Follow-up visit the next day
(J) Follow-up visit in 6 weeks
(K) Heat therapy
(L) Prescription for physiotherapy with a neurophysiological basis
(M) Diclofenac i.m. every 2 days
(N) Strict ban on bending over for 1 year
(O) CT-guided radicular infiltration
(P) Lidocaine i.m. every 2 days

7 Mrs. Zimmermann, a 73 year-old patient (1.62m, 70kg) comes to your practice at midday accompanied by her husband. Her husband reports that since his wife awoke at about 6.30am, she has been "confused and talking nonsense," which has caused him great concern. He said that yesterday everything still seemed to be normal, and upon further questioning stated that his wife had had neither a fever nor an infection, nor did she complain about having a headache. Apart from being treated for arterial hypertension (Ramipril 5mg 1-0-1) and mild hypothyroidism (L-Thyroxin 75µg 1-0-0), no other previous conditions were evident. A hysterectomy was surgically performed 15 years earlier. Mrs. Zimmermann does not drink alcohol and is a non-smoker.
Mrs. Zimmermann appears confused to you. When you request that she sits on the examination bench, she does not react. As you question Mrs. Zimmermann about her symptoms, she answers: "Yes, I can tell you that there is something wrong. But maybe not...I think one should begin at zero and not from the top. I'll tell you how it is: unlike earlier I'd first like to say something about the very big Geding-geding-ding that when I first arrived it was most important...." You then show her a pen and she calls it a "pencil", and names a watch an "arm timing device".

7.a How would you describe these symptoms? (Long Menu, 1 answer from the following list of accepted options)
Aphasia, Fluid aphasia, Wernicke's aphasia

7.b Further neurological examination identifies pronation of the right arm according to the pronator drift test, as well as slightly reduced strength in the right arm and leg. It is not possible to establish whether there are any differences in sensitivity due to the restricted communication with the patient, and you don't find any evidence for focal deficits after examination of the cranial nerves and coordination. Meningitis and fever are no present, and the results of an internistic examination such as the emergency laboratory test (blood profile, electrolytes, inflammatory/liver/kidney/thyroid values) are completely inconspicuous.
In light of the patient's history and examination results, name the 2 most probable causes of Mrs. Zimmermann's symptoms (Short Menu, 2 Answers)
(A) Benzodiazepine intoxication
(B) Herpes encephalitis
(C) Meningitis
(D) Alzheimer's disease
(E) Delirium
(F) Depression
(G) ALS (Amyotrophic Lateral sclerosis)
(H) Intracerebral bleeing
(I) Hypoglossal nerve lesion
(J) Multiple Sclerosis
(K) Brain tumour
(L) Brain metastases
(M) Cerebral ischemia

7.c In the emergency brain computer tomography (CT) that you ordered, the neuroradiologist states that there is "no evidence of fresh territorial ischemia or bleeding, or of any pronounced microangiopathic changes." The ECG shows a normal-frequency Sinus rhythm without any further conspicuous features. Ultrasound examination of the extracranial arteries reveals an 80% stenosis of the internal carotid artery on the left, and a 60% stenosis on the right. The neurological findings are still present the next morning, while Mrs. Zimmermann remains free from internistic symptoms.
What is your diagnosis now? (Long Menu, 1 answer from the following list of accepted options)
Apoplexy, Apoplexia, IIschemic Apoplexy, Ischemic stroke, Stroke

8 Ms. Hofer, a 28-year-old patient with good general health and an obese state (1,62m 84kg) was brought to the ER by ambulance. Around 45 minutes earlier, her friend had observed how Ms. Hofer suddenly fell to the floor and that her whole body twitched for 1-2 minutes. She did not react when spoken to, and her friend immediately called for an ambulance. The paramedic officer reported that she had wet herself and bitten her tongue. At this point the patient is still slightly disoriented in terms of time and space, but she responds to contact. She has no recollection of the event or of the arrival of the paramedic officers. There are no previous illnesses and the patient does not take any medication expect for the contraceptive pill. She smokes around 5-10 cigarettes a day. The patient has no experienced anything similar to this event before.
You suspect that this is a first-time general epileptic seizure. No provoking or triggering factors can be identified. However, in response to your questioning, Ms. Hofer reveals that in the last

few days she had suffered from a progressively increasing headache that was at its worst when she woke up in the morning.
The basic neurological exam (meningitis, cranial nerves, reflexes, motor function, sensitivity) shows no pathological findings. In addition, the emergency ECG and basic emergency laboratory test (limited blood profile, CRP, electrolytes, creatinine) are devoid of pathological findings and Ms. Hofer is afebrile. The results of the cranial CT that was already ordered by the ER doctors are inconspicuous

8.a What is your differential diagnosis, and which diseases need to be urgently ruled out? (Long Menu, 1 answer from the following list of accepted options)
Sinus vein thrombosis, Cerebral thrombosis

8.b You primarily suspect the patient has a sinus vein thrombosis. Which of the following diagnostic approaches would confirm/rule out your suspicions? (Long Menu, 1 answer from the following list of accepted options)
CT brain angiography-venous, Cranial MRI with venous vascular imaging , Cranial CT with venography

8 c Your suspected diagnosis of sinus vein thrombosis is confirmed by imaging results. You therefore anticoagulize the patient with unfractionated heparin.
What approach do you take to treat the epileptic seizure? (Short Menu, 1 Answer)
(A) Lorazepam i.v. 3x daily for 3 months
(B) No anti-convulsive medication in the first two weeks; after this time point begin anticonvulsive therapy if further seizures occur
(C) Emergency medication only (diazepam) if further seizures occur
(D) Perform follow-up imaging in 2 weeks; until then, slowly build up the dosage of lamotrigine (oral)
(E) No further therapy necessary, since you already treated the cause (i.e. sinus vein thrombosis)
(F) Only conservative therapy is necessary, i.e., avoid potential triggers such as sleep deprivation, excessive alcohol consumption, drugs, flickering light etc.
(G) Seizure prophylaxis with levetiracetam (oral) for 3 months, then re-evaluate
(H) Short-term seizure prophylaxis with phenytoin (oral) until anticoagulation via heparin is in the therapeutic range.

9 Mr. Bismarck, a 52-year-patient in good health with a good nutritional status (1,74m; 75kg), presents himself to the emergency ward because of severe back pain that he has experience for the last 10 days, as well as "weak feet" that developed yesterday. The referral from his general practitioner says "likely L4 syndrome," while the lumbar spine MRI results, which the patient has brought along from this morning is devoid of pathological findings. During the clinical examination you find flexor and extensor paresis (each with MRC grade 4/5) in the right foot , with a questionable sensitivity deficit at L5/S1 and a weakened Achille's tendon reflex on the right. Lasegúe's sign (straight leg raise) is negative on both sides. The remaining neurological findings from the basic examination (cranial nerve, meningitis, muscle stretch reflexes, sensory function, coordination) are inconspicuous.

9.a Which medical history details from Mr. Bismarck best help you with your further diagnostic analysis? (Short Menu, 2 Answers)
(A) Had meningitis as a child

(B) Has newly-occurring fatigue
(C) Had a partial resection of the stomach 10 years ago
(D) Had an aunt with ALS (Amyotrophic Lateral Sclerosis)
(E) Has pain mostly at night
(F) Reading is more strenuous than it used to be
(G) Has a physically demanding job
(H) Has had a vegan diet for the last two years
(I) Had stent placed in the left femoral artery 2 years ago for peripheral artery disease
(J) Has arterial hypertension, adjusted with 2-part anti-hypertensive medication.
(K) Takes regular hikes in the Black Forest

9.b Mr. Bismarck tells you that the pain especially appears at night. The emergency laboratory findings with blood cell count, coagulation factors, kidney/liver/thyroid values, and C-reactive protein are normal throughout, and the additional electrophysiological tests (EMG and sensory nerve conduction) are also yielded normal results. You suspect the patient has radiculitis. Which additional diagnostic tests are now indicated?
(Long Menu, 1 answer from the following list of accepted options)
Lumbar puncture, LP

9.c In the results of the emergency lumbar puncture you find evidence for lymphocytic pleocytosis (230/µl; normal: <5 cells/µl) and an increase in total protein (1200mg/l; normal: < 400mg/l).
What are your two main suspected diagnoses? (Long Menu, 2 answers from the following list of accepted options)
Borreliosis, Lyme disease or Lyme borreliosis, Varicella zoster virus, VZV, Borrelia burgdorferi, Neuroborreliosis, Herpes zoster

9.d In the detailed CSF analysis you find 3-class intrathecal synthesis. The VZV (varicella zoster virus)-PCR (polymerase chain reaction) is negative, and according to the cytopathological examination, there are no pathological cells. You therefore suspect Neuroborreliosis.
What are the 2 feasible treatment options? (Short Menu, 2 Answers)
(A) Ciprofloxacin i.v. for 6 months
(B) Ceftriaxon p.o. for 6 months
(C) Dexamethason i.v. for 14 days
(D) Ceftriaxon i.v. for 14 days
(E) Ciprofloxacin p.o. for 14 days
(F) No therapy necessary, since the disease is self-limiting
(G) No therapy necessary, since the infection occurred a long time ago
(H) Only symptomatic therapy necessary
(I) Doxycyclin 3x 100mg p.o for 21 days
(J) Chinin + Clindamycin p.o.
(K) Diclofenac p.o. for 14 days

10 Ms. Kirchhofer, a 21-year-old law student who is slim (1,74m; 60kg) and in good health, presents herself to your neurology practice. In the last 4 months (during which time she began her university studies and moved out of home) she has experienced episodes of dizziness that occur around 3 times a month. In addition, she would always get a strong, pulsating headache whenever she tried to study, something that she would repeatedly attempt when exams were

pending and she needed every free minute to prepare. However, when she would lie down, the dizziness and headache would disappear after 1-2 hours. She denies any hearing loss during the attacks and says that she has now come to you because she urgently has to get a grip on the situation before her exams. There are no known previous illnesses, she doesn't take any medication and has never had an operation. The MRI results that the patient has brought along does not contain any abnormalities, nor does the report from an ambulant ENT doctor, whom the patient had already visited. Your neurological examination including a nystagmus check with Frenzel glasses, head-impulse testing, and testing for skew deviation does not yield any conspicuous results.

10.a What are your 2 most probable working diagnoses? (Long Menu, 2 answers from the following list of accepted options)
Migraine, Psychological factors, Psychogenic, Psychosomatic, Somatization disorder, Vestibular migraine, Psychogenic vertigo/dizziness

10.b Which of the following details would you attach the most importance to based on your suspected diagnosis and patient history? (Short Menu, 1 Answer)
(A) Allergy to grass and pollen
(B) Drinks a maxmum of 1-2 beers at the weekend
(C) Smoked cannibus between the ages of 16 and 18
(D) Smokes half a packet of cigarettes a day
(E) Was in India 3 months ago
(F) Has a pet bird
(G) Has had diffuse joint pain for the last few weeks
(H) Has had mild diarrhea for the last 3 days
(I) Positive family history for breast cancer (grandmother)
(J) Positive family history for heart attack (grandfather)
(K) Positive family history for schizophrenia (uncle)
(L) Positive family history for migraine (mother)
(M) Weight loss of 1-2 kg in the last 6 months
(N) Has lacked energy and felt defeated over the last 2 months
(O) Temperature of 37.6°C

10.c The patient reports that her mother suffered from severe migraines between the ages of 20 and 40. You establish a diagnosis of vestibular migraine in Ms. Kirchhofer.
Should an attack recur, what do you give the patient for acute treatment? (Short Menu, 2 Answers)
(A) Acetylsalicylic acid 1000mg
(B) Acetylsalicylic acid 100mg
(C) Valproate
(D) ß-blockers
(E) Magnesium
(F) Triptane
(G) Paracetamol 125mg
(H) Dexamethasone 1000mg

10.d You have prescribed the patient with ASS 1000mg as well as Triptane when required, and ask her to keep a migraine diary, with which she should present to you again in 2 months.

At this follow-up appointment, Ms. Kirchhofer reports that although the medication has been helpful, she is once again having between 3-5 attacks per month. You now want to introduce additional prophylactic medication.
Which of following are worth considering? Choose 2 answers!
(Short Menu, 2 Answers)
(A) Propanolol
(B) Lidocaine
(C) Phenytoin
(D) Dihydroergotamin
(E) Metoprolol
(F) Prednisone
(G) Amitriptyline
(H) ACE-Hemmer
(I) Pregabaline
(J) Clobazam
(K) Citalopram
(L) Acetylsalicylic acid

11 Mr Best is a 33-year-old patient in a reduced general health state and cachectic condition (1.85m; 67kg). He is taken to the ER by a friend who is worried about him, and claims that Mr. Best has changed since this morning and is intermittently confused. Mr. Best himself says that "he doesn't feel well." He is awake, oriented to person and place, but unsure about the situation ("doctor's office") and time ("10th January instead of the 17th). Mr. Best seems nervous and restless. You notice a fine tremor, and that his hands are markedly sweaty. His blood pressure is elevated to 180/100 mmHg, and the monitor shows a sinus tachycardia of 112/min. The blood glucose level is 98 mg/dl. Mr. Best is afebrile. The rest of the medical exam is normal. The neurological exam does not reveal any hint of a focal deficit. Mr. Best reports no previous illnesses or operations, and does not take any regular medication. The ER file shows 4 previous stays in the past 6 months because of alcohol intoxication. When asked, Mr Best assures you that he has not drank any alcohol or take any drugs in the past 2 days. This fits with the blood alcohol level of 0.0 g/l. Apart from slightly-raised hepatic parameters, the remaining lab parameters are normal.

11.a Name the two most likely suspected diagnoses (Long Menu, 1 answer from the following list of accepted options)
Alcohol withdrawal syndrome, Withdrawal syndrome

11.b When taking the extended history and asking about alcohol consumption, Mr Best claims to have been "sober" for 2 days (he claims to have otherwise consumed 8-10 bottles of beer per day for the last 2 years) and now "finally wants to stop drinking alcohol". Again, he denies taking any further drugs. You suspect alcohol withdrawal syndrome. You want to admit Mr Best for further treatment.
What are the necessary pharmacological measures in this case? Name two drugs from different substance classes. (Long Menu, 2 answers from the following list of accepted options)
Clomethiazol (e.g., Distraneurin®) (Sedativa), Diazepam (e.g., Valium®, Valiquid®) (Benzodiazepine), Lorazepam (e.g., Tavor®) (Benzodiazepine), Vitamin B1 (e.g., Betabion®)

11.c Mr Best leaves the ER shortly thereafter against medical advice. His friend brings him back 10 hours later in markedly worse condition. Mr Best is agitated and disoriented, but still

able to communicate with you. He reports to being able to "see white mice". His blood pressure is measured at 200/120 mmHg and he has a heart rate of 125/min. Which of the following therapeutic options is best in this case? (Short Menu, 1 Answer)
(A) Intensive care admission, vitamin B12, clomethiazole, haloperidole and carbamazepine
(B) Intensive care admission, vitamin B1, clomethiazole and carbamazepine
(C) Intensive care admission, vitamin B12, haloperidole and carbamazepine
(D) Intensive care admission, vitamin B1, levetiracetam and clonidine
(E) Intensive care admission, vitamin B12, clonidine and haloperidole
(F) Intensive care admission, vitamin B1, clomethiazole, haloperidole and clonidine
(G) Discharge home, vitamin B12, clomethiazole, haloperidole and carbamazepine
(H) Discharge home, vitamin B1, clomethiazole and carbamazepine
(I) Discharge home, vitamin B12, haloperidole and carbamazepine
(J) Discharge home, vitamin B1, levetiracetam and clonidine
(K) Discharge home, vitamin B12, clonidine and haloperidole
(L) Discharge home, vitamin B1, clomethiazole and haloperidole
(M) General ward admission, vitamin B12, clomethiazole, haloperidole and carbamazepine
(N) General ward admission, vitamin B1, clomethiazole and carbamazepine
(O) General ward admission, vitamin B12, haloperidole and carbamazepine
(P) General ward admission, vitamin B1, levetiracetam and clonidine
(Q) General ward admission, vitamin B12, clonidine and haloperidole
(R) General ward admission, vitamin B1, clomethiazole and haloperidole

12 Karl Marwick, a 20-year-old patient, is brought to the ER by ambulance officers. His girlfriend reported that she saw Mr. Marwick lying on the couch and "hitting himself", and has foam on his mouth. At that point she called the emergency service, whose officers brought Mr. Marwick to your ER. Mr. Marwick is awake upon arrival, but has no recollection of the event. He has not experienced anything similar in the past. You then question his girlfriend further in an attempt to learn more details about the acute episode.

12.a Which of the following options would most likely point to the occurrence of an epileptic seizure?
(Short Menu, 3 Answers)
(A) Eyes open, gazed look
(B) Duration of 5-10 min
(C) Slack body tone
(D) Eyes squinted
(E) Duration of >10 min
(F) Duration of 1-2 min
(G) Eyes open and rolled back
(H) Lateral tongue bite
(I) Reorientation after a few seconds

12.b The basic neurological exam (meningitis, cranial nerves, motor function, sensitivity, and reflexes) is devoid of pathological findings. What do you order (emergency-wise) as the next diagnostic test? (Long Menu, 1 answers from the following list of accepted options)
Laboratory test, Blood test

12.c The emergency lab values from Mr. Marwick are completely inconspicuous. In response to your question, Mr. Marwick reports that he did not notice an infection with fever or a strong

headache before the seizure, and his girlfriend confirms that he had been "his usual self." You explore possible aggravating factors. Which of the following points could have provoked an epileptic seizure?
(Short Menu, 1 Answer)
(A) A relatively low fluid intake (~1.5 litre) in the last 24 hours
(B) The only things consumed in the last 24 hours were breakfast and a piece of pizza
(C) No alcohol consumption in the last ten days ago due to the onset of a fasting period [otherwise one bottle of beer (0.33 l) per day in the past 6 months]
(D) Excessive tobacco consumption (> 30 cigarettes per day)
(E) Regular cannabis consumption until 2 years ago (1-2 joints every two weeks)
(F) Once-off amphetamine consumption (speed) around 6 months ago
(G) Intake of an opioid ((tilidine + naloxone) for lumbar pain for the last 2 weeks.
(H) Only slept for 6 hours last night (usually 7-8 hours per night)
(I) Unusually high level of physical activity (2 hours of soccer) the day before
(J) None of the above

12.d Based on Mr. Marwick's details, which of the following steps and recommendations are necessary for the diagnostics, treatment and assessment of his driving ability?
(Short Menu, 3 Answers)
(A) Discharge without further clarification, since this deals with an occasional convulsion
(B) Send the patient home with the recommendation for further clarification in an outpatient clinic (EEG, cMRT)
(C) Emergency admission to the ICU for monitoring
(D) Emergency contrast-enhanced CCT (cranial computer tomography) in the ER
(E) Emergency lumbar puncture in the ER
(F) No specific therapy, ban of endurance sports
(G) No anticonvlusive medication for the time being, just provide education on the avoidance of provoking/triggering factors
(H) Immediate initiation of long-term therapy with valproic acid
(I) immediate administration of high-dose lorazepam i.v. on the intensive care unit
(J) Immediate initiation of a slow lamotrigine up-titration
(K) Inform the patient about the details of the 12-month driving ban
(L) Driving ban until the necessary blood levels of the anticonvulsive drugs are reached
(M) Revocation of his driver’s license due to the diagnosis of epilepsy
(N) Inform the patient about the details of the 6-month driving ban
