# Supplementary material for: Validation and perception of a key feature problem examination in neurology
Source: PLoS One. 2019 Oct 18;14(10):e0224131. doi: 10.1371/journal.pone.0224131 (PMC6799971; doi:10.1371/journal.pone.0224131)
Supplement: S2 File — (PDF) [file pone.0224131.s002.pdf]

Key Feature Problem Examination (German version)

1 Herr Heinrich, ein 74-jähriger Patient in leicht reduziertem Allgemeinzustand und normalen Ernährungszustand (1,78m, 78kg), stellt sich wegen seit 3-4 Wochen bestehender Kopfschmerzen und jetzt deutlich zunehmender Schläfrigkeit in der Notaufnahme vor. Die begleitende Ehefrau berichtet, ihr Mann sei früher ein lebenslustiger, aktiver Mensch gewesen. Jetzt sei er aber öfter apathisch, würde immer wieder einfach so einschlafen und sei auch irgendwie „vom ganzen Wesen nicht mehr so wie früher". An Vorerkrankungen sind eine TIA (Transitorische ischämische Attacke) 2008, chronisches Vorhofflimmern und eine arterielle Hypertonie bekannt. Herr Heinrich gibt an, seit 10 Jahren keinen Alkohol mehr zu trinken und seit 20 Jahren auch nicht mehr zu rauchen. An Medikamenten nimmt er zur Blutdrucksenkung Ramipril 2,5mg 1-0-1 und zur Antikoagulation Phenprocoumon (Marcumar®) (Ziel-INR (International Normalized Ratio)-Wert zwischen 2,0 und 3,0). Internistisch zeigt sich ein bis auf das bekannte Vorhofflimmern unauffälliger Befund, es ergibt sich kein Hinweis auf einen Infekt, Herr Heinrich ist afebril.
In der neurologischen Untersuchung ist Herr Heinrich schläfrig, aber stets kontaktbar. Er wirkt insgesamt verlangsamt und kann sich schlecht konzentrieren. Sie finden außer einer subjektiven Schwere des linken Arms im Vorhalteversuch mit einer leichten Ungeschicklichkeit der linken Hand keine motorischen oder sensiblen Defizite, der Hirnnervenbefund ist regelgerecht, es zeigt sich kein Meningismus. Beim Strichgang zeigt sich eine leichte Tendenz nach links abzuweichen, im Romberg zeigt sich ebenfalls eine Tendenz nach links zu kippen.
Das Notfall-Labor zeigt einen INR-Wert von 2,84. Die anderen Werte (Blutbild, Elektrolyte, Entzündungs-, Leber, Nieren- und Schilddrüsenwerte) sind ohne pathologischen Befund.

1.a Was ist Ihre Haupt-Verdachtsdiagnose?
(Short Menu, 1 Antwort)
(A) Transiente globale Amnesie (TGA)
(B) Pharmakogene Ursache (Ramipril und Phenprocoumon)
(C) Erstdiagnose einer Epilepsie
(D) Chronischer Alkoholabusus trotz anders lautender eigener Angabe
(E) Erstdiagnose einer Demenz
(F) Intrakranielle Raumforderung
(G) Ischämischer Schlaganfall
(H) Encephalitis
(I) "Parainfektiöse" Enzephalopathie

1.b Sie wollen eine intrakranielle Raumforderung ausschließen und veranlassen eine zerebrale Bildgebung. Im CCT sehen Sie eine sichelförmige, zum Hirn konkav verlaufende Raumforderung (mit iso-, hyper- und hypodensen Anteilen) zwischen Kortex und Schädelkalotte von doppelter Kalottenbreite und eine Mittellinienverlagerung des Gehirns von 7mm.
Welche Diagnose stellen Sie nun? (Short Menu, 1 Antwort))
(A) Akute Subarachnoidalblutung
(B) Akutes Epiduralhämatom
(C) Akutes Subduralhämatom
(D) Akute Meningitis
(E) Akute Encephalitis

(F) Einblutung bei parieto-frontalem Meningeom
(G) Glioblastom
(H) Medulloblastom
(I) Chronische Subarachnoidalblutung
(J) Chronisches Epiduralhämatom
(K) Chronisches Subduralhämatom
(L) Chronische Meningitis
(M) Chronische Encephalitis
(N) Meningeosis neoplastica

1.c Sie stellen die Diagnose eines chronischen Subduralhämatoms mit beginnenden fokalneurologischen Defiziten bei deutlich raumforderndem Effekt.
Was wäre der nächste therapeutische Schritt? (Long Menu, 1 Antwort aus der folgenden Liste der akzeptierten Möglichkeiten)
Bohrlochtrepanation des Schädels, Entleerung eines chronischen subduralen Hämatoms ggf. mit Drainage, Operation, Operation: Ausräumung eines subduralen Hämatoms, Operation eines subduralen Hämatoms

2 Herr Rudolf, ein 58-jähriger Patient in gutem Allgemeinzustand und leicht adipösen Ernährungszustand (1,78m; 90kg) stellt sich in der Notaufnahme wegen seit 3 Stunden bestehender, starker Rückenschmerzen und neu aufgetretener Gehschwäche ("Stolpern") vor. Er habe zwar häufig Rückenschmerzen, diesmal seien sie aber „besonders schlimm“. An eine besondere Auslöse-Situation oder ein Trauma kann er sich nicht erinnern. Beruflich ist er in der Bau-Branche tätig. Er ist verheiratet und hat 2 erwachsene Kinder. Er ist Nichtraucher. An Vorerkrankungen ist ein arterieller Hypertonus bekannt, eine Operation hatte er schon wegen einer Leistenhernie, und eine Tosillektomie.
In der klinischen Untersuchung finden Sie eine Fußheber- und Fußsenker-Parese mit Kraftgrad 4/5, die rechts leicht stärker ausgeprägt ist als links, ein positives Lasègue-Zeichen beidseits, sowie eine Hypästhesie am rechten Schienbein und der gesamten rechten Bein-Rückseite sowie beidseits an den Gesäßhälften. Der Achillessehnenreflex ist beidseits ausgefallen bei unauffälligen sonstigen Muskeleigenreflexen. Das Lasegúe-Zeichen ist beidseits positiv. Die restliche neurologische Basis-Untersuchung (Hirnnerven, Meningismus) ist ohne pathologischen Befund. Auf die Frage nach Wasserlassen und Stuhlgang gibt Herr Rudolf an, in den letzten 3 Stunden nicht auf Toilette gemusst zu haben.

2.a Welche klinischen Zusatz-Untersuchungsschritte führen Sie daraufhin noch durch? (Long Menu, 1 Antwort aus der folgenden Liste der akzeptierten Möglichkeiten)
Analreflex, Cremasterreflex, Bulbocavernosus Reflex, Sphinktertonus, Analsphinktertonus

2.b Sie finden zusätzlich zum oben genannten Untersuchungsbefund einen verminderten Analsphinktertonus, sowie einen rechtsseitig abgeschwächten Analreflex.
Was ist nun ihre Verdachtsdiagnose? (Long Menu, 1 Antwort aus der folgenden Liste der akzeptierten Möglichkeiten)
Cauda-equina-Syndrom, Cauda-Syndrom, Kauda-equina-Syndrom, Kauda-Syndrom, Conus-Cauda-Syndrom, Konus-Kauda-Syndrom

2.c Was wäre der nächste apparative diagnostische Schritt (Zusatzdiagnostik)? (Long Menu, 1 Antwort aus der folgenden Liste der akzeptierten Möglichkeiten)

MRT lumbal, Magnetresonanztomographie lumbal, Magnetresonanztomographie der LWS, MRT der LWS, CT der LWS, CT-lumbal, Computertomographie-lumbal, Computertomographie der LWS,

2.d Im MRT der LWS finden Sie einen großen medialen Bandscheibenvorfall mit Kompression der Kauda-Fasern.
Was ist der nächste Therapieschritt? (Short Menu, 1 Antwort)
(A) Schmerzreduktion mit Diclofenac p.o.
(B) Reha-Antrag vorschlagen, da eine Rehabilitationsbedürftigkeit vorliegt
(C) Überweisung zum Orthopäden
(D) Überweisung zum ambulanten Neurologen zur Verlaufskontrolle
(E) Watch and wait
(F) Erneute Vorstellung in 2 Wochen
(G) Schmerzreduktion mit lokal Lidocaoin s.c.
(H) Notfall-Operation
(I) Schmerzreduktion mit Diclofenac i.m.
(J) Verschreibung von Physiotherapie
(K) Überweisung zum Onkologen zur weiteren Abklärung
(L) Lokal Fentanyl-Pflaster
(M) Überweisung zum ambulanten Neurochirurgen zur Operations-Planung
(N) Geplante Operation in 3-4 Tagen

3 Frau Hackmann, eine 62-jährige Patientin in gutem Allgemeinzustand und leicht adipösen Ernährungszustand (1,62m; 81kg) stellt sich bei Ihnen in der Praxis vor. Seit 2 Tagen träte insbesondere beim Aufstehen aus dem Bett oder bei schnellen Bewegungen heftiger Drehschwindel auf. Sie müsse sich dann sofort hinlegen und die Augen schließen, dann würde es rasch besser werden. Während des Schwindels würde es ihr auch übel werden. In den Phasen zwischen den Schwindelattacken bemerke Frau Hackmann keine sonstigen Defizite. Als Vorerkrankungen gibt sie eine arterielle Hypertonie und eine Hypothyreose an. An Voroperationen hat sie eine Appendektomie und eine Hysterektomie.

3.a Welche 2 Fragen helfen Ihnen für die Schwindeldiagnostik am meisten weiter?
(Short Menu, 2 Antworten)
(A) Provozierbar durch Bewegungen?
(B) Dauer der Attacken?
(C) Nachtschweiß?
(D) Durchfall?
(E) Kopfschmerzen?
(F) Fieber?
(G) Gewichtsabnahme?
(H) Familienanamnese?
(I) Psychiatrische Vorerkrankungen?
(J) Besserung durch Sport?
(K) Allergien?
(L) Lichtempfindlichkeit?
(M) kognitive Beeinträchtigungen?
(N) Sozialanamnese?
(O) Schmerzen?
(P) Auslandsaufenthalte?

3.b Auf detailliertes Nachfragen erzählt die Patientin Ihnen, dass der Drehschwindel vor allem bei schneller Kopfdrehung nach rechts auftrete und nach etwa 1-2 Minuten im Liegen wieder vollständig abklingen würde. Sie denken daher an einen Benignen Paroxysmalen Lagerungsschwindel (BPLS)
Sie führen eine neurologische Basis-Untersuchung (Meningismus, Hirnnerven, Muskeleigenreflexe, Sensibilität, Motorik, Koordination) durch.
Welcher der folgenden Untersuchungsbefunde spricht gegen Ihre Verdachtsdiagnose? (Short Menu, 1 Antwort)
(A) Leichter Rigor rechter Arm
(B) Eudiadochokinese beidseits
(C) Negativer Romberg-Versuch
(D) Negativer Unterberger-Tretversuch
(E) Finger-Nase-Versuch beidseits sicher
(F) Vibrationsempfinden 5/8 an beiden Großzehen-Grundgelenken
(G) Erschöpflicher Blickrichtungsnystagmus
(H) Bizeps-Sehnen-Reflex links leicht abgeschwächt
(I) Unauffällige Innervation des N.facialis beidseits
(J) Zeitliche Orientierung leicht unscharf (24. statt 26. November)
(K) Positiver Kopfimpulstest nach rechts
(L) Hypästhesie an der linken Hand-Innenfläche
(M) Kraftgrad 4-5/5 des linken Fußhebers
(N) Bauchhaut-Reflexe beidseits nicht auslösbar
(O) Innervation des N. hypoglossus beidseits regelgerecht
(P) Kopfimpuls-Test negativ
(Q) Erhöhte Wendeschrittzahl (10 Schritte)

3.c Frau Hackmann berichtet auf Ihre Nachfrage, dass sie vor einigen Monaten ein paar Tage lang heftigen Schwindel mit Übelkeit und Erbrechen gehabt hätte, nachdem es sich aber nach ein paar Tagen von selbst komplett gebessert hätte, wäre sie nicht mehr zum Arzt gegangen.
Was ist Ihr nächster diagnostischer Schritt bezüglich der aktuellen Schwindelattacken (Long Menu, 1 Antwort aus der folgenden Liste der akzeptierten Möglichkeiten)
Lagerung, Lagerungsprobe nach Dix-Hallpike, Lagerungsmanöver, Lagerungsprobe, Lagerungsprobe nach Hallpike

3.d Ihre Verdachtsdiagnose eines benignen peripheren Lagerungsschwindel bestätigt sich, da Sie in der Lagerungsprobe nach rechts einen Nystagmus zum unten liegenden Ohr mit rotatorischer Komponente und begleitendem Drehschwindel, der weniger als eine Minute andauert, finden. Sie führen mit der Patientin das therapeutische Lagerungs-Manöver nach Semont erfolgreich durch und Frau Hackmann berichtet in der erneuten Vorstellung nach einer Woche, dass Ihre Beschwerden seitdem verschwunden seien.
Nach 4 Monaten kommt sie notfallmäßig erneut mit den gleichen 1-2 minütigen Schwindelattacken in Ihre Praxis. Symptomatik. Was ist nun Ihre erste Verdachtsdiagnose? (Long Menu, 1 Antwort aus der folgenden Liste der akzeptierten Möglichkeiten)
Benigner paroxysmaler Lagerungsschwindel, BPLS, Benigner paroxysmaler Lagerungsschwindel-Rezidiv, BPLS-Rezidiv

4 Herr Maier, ein 58-jähriger Lehrer (1,80m; 98kg) wird Ihnen in der Notaufnahme vorgestellt. Vor 3 Stunden sei er von Passanten in der Stadt auf dem Boden liegend und „zuckend“ gesehen worden. Diese hätten dann den Notruf alarmiert. Bei Eintreffen des Rettungsdienstes zeigten sich normale Vitalparameter (Blutdruck 128/78 mmHg, Herzfrequenz 88/min), der Blutzucker betrug 134 mg/dl. Herr Maier ist jetzt wieder wach und voll orientiert, jedoch immer noch deutlich verlangsamt. An das Zucken und dass er gefallen sei könne er sich nicht erinnern. Die letzten Tage habe er sich sehr angeschlagen und erkältet gefühlt, und auch einmalig Erbrochen. Zudem habe er seit einigen Tagen starke Kopfschmerzen. An Vorerkrankungen ist ein diätetisch eingestellter Diabetes mellitus Typ 2 bekannt. Sie führen eine neurologische Untersuchung durch.

4.a Welchem der folgenden Befunde müssen Sie besondere Beachtung schenken? (Short Menu, 1 Antwort)
(A) Pallhypästhesie 5/8 der Großzehen-Grundgelenke beidseits
(B) Verlangsamte und fehlerhafte serielle Subtraktion (serial seven), Rückwärtsbuchstabieren von RADIO fehlerhaft.
(C) ASR (Achillessehnenreflex) beidseits abgeschwächt
(D) Trömner-Reflex beidseits negativ
(E) Negativer Palmomentalreflex
(F) Glabellareflex erschöpflich
(G) Erschöpflicher Endstellungsnystagmus beidseits
(H) Leicht vermindertes Hörvermögen rechts
(I) Beidseitiges Fehlen der Bauchhautreflexe

4.b Aufgrund des pathologischen Befundes der neuropsychologischen Bedside-Untersuchung schließen Sie auf eine reduzierten Konzentrations- und Aufmerksamkeitsleistung bei Herrn Maier. Die sonstigen Befunde der neurologischen Untersuchung (Meningismus, Hirnnerven, Motorik, Reflexe, Sensibilität) sind ohne pathologischen Befund. Die aktuellen Vitalparameter in der Notaufnahme sind nun: Puls 92/min, Blutdruck 141/82mmHg, Temperatur 38,8°C, Blutzucker 121mg/dl.
Was ist nun Ihre Verdachtsdiagnose? (Long Menu, 1 Antwort aus der folgenden Liste der akzeptierten Möglichkeiten)
Herdenzephalitis, Encephalitis, Enzephalitis

4.c Sie haben den Verdacht einer Enzephalitis. Dazu passend zeigen sich in den Laborwerten eine Leukozytose mit 13.000 Leukozyten/µl (Norm: 3.800-10.500/µl) und ein C-reaktives-Protein (CRP) von 48mg/l (Norm: <10mg/l). Sie nehmen daraufhin Blutkulturen ab. Was unternehmen Sie diagnostisch als nächstes? Nennen Sie Ihre nächsten 2 Schritte! (Long Menu, 2 Antworten aus der folgenden Liste der akzeptierten Möglichkeiten)
Liquorpunktion, LP, Lumbalpunktion, Punktion-lumbal, Magnet-Resonanz-Tomographie-cranial, MRT-cranial, NMR-kranial, Kernspinresonanz-kranial, cMRT, Zerebrale Schnittbildgebung, Cerebrale Schnittbildgebung, Zerebrale Schnittbildgebung mit Kontrastmittel, Cerebrale Schnittbildgebung mit Kontrastmittel, CCT mit Kontrastmittel, CT-kranial mit Kontrastmittel, Computer-Tomographie-cranial mit Kontrastmittel, PCR aus dem Liquor, Polymerase-Kettenreaktion aus dem Liquor, CCT, CT-kranial, Computer-Tomographie-cranial

4.d In der Liquordiagnostik ergibt sich eine Zellzahl von 189/µl (Norm: < 4/µl) und ein Gesamteiweiß von 69 mg/100ml (Norm: < 40mg/100ml). Im cMRT finden Sie eine

hyperintense Diffusionsstörung temporal links. Was ist der wahrscheinlichste Erreger? (Long Menu, 1 Antwort aus der folgenden Liste der akzeptierten Möglichkeiten)
Herpes simplex Typ 1

5 Herr Hofrichter, ein 46-jähriger Mann in gutem Allgemeinzustand und schlanken Ernährungszustand (1,82; 84kg) stellt sich in Ihrer Praxis vor. Seit einem Tag habe er durchgehend leichten Drehschwindel und Übelkeit und hätte auch einmalig Erbrochen. Er könne seitdem seinem Beruf (Informatiker) nur schlecht nachgehen, da er die Steuerung mit der Computer-Maus kaum noch ausüben könne. Er bemerke auch ein unsicheres Gehen. Medikamente nehme er keine, Vorerkrankungen sind ebenfalls keine bekannt. An Operationen hatte er schon eine Sprunggelenks-Fraktur (Weber B). Das von Ihrer Sprechstundenhilfe bereits angefertigte EKG zeigt einen normofrequenten Sinusrhythmus.

5.a Was fragen Sie den Patienten als nächstes? Markieren Sie die wichtigste Frage! (Short Menu, 1 Antwort)
(A) Begleitender Tinnitus?
(B) Epigastrische Aura?
(C) Zeitlicher Verlauf des Auftretens?
(D) Psychiatrische Vorerkrankungen?
(E) Paniksymptome?
(F) Lichtempfindlichkeit?
(G) Provozierbar durch Bewegungen?
(H) Situationsabhängige Verbesserung bzw. Verschlechterung?
(I) Besserung durch Sport?
(J) Gewichtsabnahme?
(K) Allergien?
(L) Berufliche Belastungssituationen?
(M) Polyurie und Polydypsie?

5.b Herr Hofrichter berichtet, dass der Schwindel mittags ganz plötzlich innerhalb von einer Minute begonnen habe und seitdem im Wesentlichen unverändert vorhanden sei. Eine Lageabhängigkeit verneint er, wenngleich bei allen Lageänderungen eine vermehrte Übelkeit aufträte. Als sie sich länger mit ihm unterhalten, meinen Sie eine leichte Dysarthrie feststellen zu können, sind sich aber aufgrund des Dialektes des Patienten nicht sicher. Des Weiteren fällt Ihnen ein geringgradiger Upbeat-Spontannystagmus auf.
Welche der folgenden Untersuchungsschritte sind in diesem Fall besonders wichtig bzw. für Ihre weitere Diagnostik zielführend?
Markieren Sie 7 Antworten!
(Short Menu, 7 Antworten)
(A) Gangbild prüfen
(B) Zeigeversuche (Finger-Nase und Knie-Hacke) prüfen
(C) Meningismus prüfen
(D) Diagnostische Lagerungsprobe durchführen
(E) Lhermitte-Zeichen prüfen
(F) Diadochokinese-Prüfung
(G) Kopfimpuls-Test
(H) Vibrationsempfindungs-Prüfung
(I) Romberg-Versuch
(J) Testung auf Skew deviation

(K) Muskeleigenreflexe untersuchen
(L) Fremdreflexe untersuchen
(M) Zungenbeweglichkeit untersuchen
(N) Epikritsche Sensibilität prüfen
(O) Rigorprüfung
(P) Würgereflex-Testung
(Q) Korneal-Reflex-Testung
(R) Tiefensensibilität-Prüfung
(S) Schmerzempfindungs-Prüfung
(T) Nystagmusprüfung mit Frenzelbrille

5.c Sie finden in der neurologischen Untersuchung eine Standunsicherheit und Fallneigung nach rechts (im Romberg-Versuch schon bei geöffneten Augen), sowie eine Zeigeataxie rechts. Der Upbeat-Nystagmus bestätigt sich unter der Frenzelbrille, der Kopfimpulstest ist beidseits negativ.
Was führen Sie nun als weitere apparative Diagnostik als erstes durch? (Long Menu, 1 Antwort aus der folgenden Liste der akzeptierten Möglichkeiten)
Magnet-Resonanz-Tomographie-cranial, Kernspinresonanz-kranial, NMR-kranial, MRT-cranial, cMRT, Cerebrale Schnittbildgebung, Zerebrale Schnittbildgebung, CCT ohne Kontrastmittel, CT-kranial ohne Kontrastmittel, Computer-Tomographie-cranial ohne Kontrastmittel

5.d In der cMRT finden Sie einen Kleinhirninfarkt rechts, der ca. 2/3 der Kleinhirnhemisphäre betrifft, bei zugrundeliegendem Verschluss der A. vertebralis rechts aufgrund eines Dissekats. Sie beginnen eine Sekundärprophylaxe mit ASS 100mg.
Am nächsten Morgen ist Herr Hofrichter zunächst zunehmend agitiert, wird dann im Verlauf aber zunehmend schläfrig. Es zeigen sich unauffällige Vitalparameter sowie ein unauffälliger Laborbefund.
An welche schwerwiegende neurologische Komplikation müssen Sie nun bei diesem Krankheitsbild unbedingt denken? (Long Menu, 1 Antwort aus der folgenden Liste der akzeptierten Möglichkeiten)
Hirnödem, Hirnstammkompression, Hydrocephalus occlusus, Einklemmung, Infratentorielle Einklemmung, Sekundäre Einblutung, Liquoraufstau, Aufstau von Liquor

6 Herr Winkelmann, ein 45-jähriger Patient in gutem Allgemeinzustand und normalen Ernährungszustand (1,82m; 80kg) kommt in die Notaufnahme wegen seit gestern bestehenden, starken Rückenschmerzen mit Ausstrahlung in das rechte Bein. Die Schmerzen seien ganz plötzlich beim Heben eines Wasserkastens aufgetreten. Vorerkrankungen sind keine bekannt, Medikamente nimmt er ebenfalls keine, an Operationen hatte er schon eine Appendektomie.
Bei der neurologischen Basisuntersuchung (Meningismus, Hirnnerven Muskeleigenreflexe, Motorik, Sensibilität, Koordination) finden Sie außer einer leichten Hypästhesie an der rechten Unterschenkelvorderseite sowie eine Parese der Fußheber mit Kraftgrad 4/5 rechtseitig einen unauffälligen Befund.

6.a Welche weiteren Zusatz-Untersuchungsschritte helfen Ihnen in diesem Fall bei der Differentialdiagnose Radikulopathie versus Neuropathie? (Long Menu, 2 Antworten aus der folgenden Liste der akzeptierten Möglichkeiten)
Trendelenburg-Zeichen, Laségue-Zeichen

6.b Sie finden ein positives Lasègue-Zeichen rechts und ein positives Trendelenburg-Zeichen rechts. Wo vermuten Sie die Läsion? (Short Menu, 1 Antwort)
(A) L5 rechte Wurzel
(B) N.peroneus rechts
(C) N.peroneus links
(D) N.tibialis rechts
(E) N.tibialis links
(F) Tractus corticospinalis (Pyramidenbahn)
(G) Capsula interna
(H) L5 linke Wurzel
(I) L4 rechte Wurzel
(J) L4 linke Wurzel
(K) Motorische Endplatte
(L) Musculus tibialis anterior
(M) Musculus extensor hallucis longus
(N) Musculus gastrocnemicus
(O) thorakales Myelon rechts

6.c Sie vermuten eine Radikulopathie der Wurzel L5 rechts.
Welche weitere Diagnostik ist nun zwingend notwendig? (Short Menu, 1 Antwort)
(A) Lumbalpunktion
(B) MRT (Magnetresonanztomographie) der LWS
(C) CT (Computertomographie) der LWS
(D) EMG (Elektromyographie)
(E) NLG (Nervenleitgeschwindigkeit)
(F) keine weitere Diagnostik notwendig
(G) Röntgen LWS
(H) Myelographie
(I) PET (Positronen-Emissionscomputertomographie)
(J) Funktionsaufnahmen der LWS
(K) Messung der Knochendichte
(L) Liquorszintigraphie
(M) SSEP (somatosensibel evozierte Potenziale)
(N) Biopsie

6.d Sie verordnen Diclofenac 75mg 2xtgl mit begleitendem Magenschutz. Am übernächsten Tag stellt sich der Patient bei Ihnen erneut mit deutlich gebessertem Beschwerdebild vor. Was ist nun das beste Procedere? (Short Menu, 3 Antworten)
(A) Überweisung zum Orthopäden
(B) sofortige Wiedervorstellung bei Verschlechterung der Paresen oder Auftreten von Blasen-/ Mastdarmstörungen empfehlen
(C) Schnellstmögliche Operation
(D) Überweisung zur elektiven Operation
(E) Weiter Diclofenac p.o. für 1-2 Wochen
(F) Vorstellung beim Neurochirurgen
(G) Stationäre Einweisung
(H) Strikte Stufenbettlagerung für 2 Wochen
(I) Wiedervorstellung morgen

(J) Wiedervorstellung in 6 Wochen
(K) Wärmetherapie
(L) Verordnung von Physiotherapie auf neurophysiologischer Grundlage
(M) Alle 2 Tage Diclofenac i.m.
(N) Striktes Verbot von Bückbewegungen für 1 Jahr
(O) CT-gesteuerte Wurzel-Infiltration
(P) Alle 2 Tage Lidocain i.m.

7 Frau Zimmermann, eine 73-jährige Patientin (1,62m, 70kg) kommt mit ihrem Ehemann um 12 Uhr in Ihre Praxis. Der Ehemann berichtet, dass sie seit dem Aufwachen um ca. 6:30 „verwirrt sei und wirr rede“, er mache sich nun große Sorgen. Gestern sei noch alles normal und wie immer gewesen. Auf Nachfrage gibt er an, dass seine Frau in den letzten Tagen weder Fieber oder einen Infekt gehabt hätte noch über Kopfschmerzen geklagt hätte. Vorerkrankungen sind außer einer arteriellen Hypertonie (behandelt mit Ramipril 5mg 1-0-1) und einer leichten Hypothyreose (behandelt mit L-Thyroxin 75µg 1-0-0) keine bekannt. An Operationen erfolgte eine Hysterektomie vor 15 Jahren. Frau Zimmermann trinke keinen Alkohol und rauche nicht.
Frau Zimmermann wirkt auf Sie verwirrt. Auf die Bitte hin, sich auf Ihre Untersuchungsliege zu setzen, reagiert sie nicht. Als Sie Frau Zimmermann nach ihren Beschwerden fragen, antwortet sie: "Ja das kann ich Ihnen sagen, dass ich Beschwerden habe. Na ich muss mal anders... Ich glaube man sollte bei Null beginnen und nicht bei oben. Es ist so: Gegenüber früher möcht ich erst einmal sagen
über den ganz großen Geding-geding-ding erstmal als ich ankam ist es natürlich ganz entschieden...".. Den von Ihnen gezeigten Kugelschreiber nennt Sie „Bleistift“, eine Armbanduhr „Armzeitmessgerät“.

7.a Wie würden Sie dieses Symptom bezeichnen? (Long Menu, 1 Antwort aus der folgenden Liste der akzeptierten Möglichkeiten)
Aphasia, Aphasie, Flüssige Aphasie, Wernicke-Aphasie

7.b In der weiteren neurologischen Untersuchung stellen Sie ein Pronation des rechten Armes im Vorhalteversuch sowie eine minimal reduzierte Kraft im rechten Arm und Bein fest. Sensibilitätsunterschiede können Sie aufgrund der eingeschränkten Kommunikation nicht beurteilen, sie finden keine Hinweise auf fokale Defizite bei der Untersuchung der Hirnnerven und der Koordination. Es besteht kein Meningismus oder Fieber, sowohl die internistische Untersuchung wie auch das Notfalllabor (Blutbild, Elektrolyte, Entzündungs-, Leber-, Nieren- und Schilddrüsenwerte) sind komplett unauffällig.
Nennen Sie in Zusammenschau mit der Anamnese und Untersuchungsbefund die 2 wahrscheinlichsten Ursachen für Frau Zimmermanns Symptome! (Short Menu, 2 Antworten)
(A) Benzodiazepin-Intoxikation
(B) Herpes Enzephalitis
(C) Meningitis
(D) Morbus Alzheimer
(E) Delir
(F) Depression
(G) ALS (Amyotrophe Lateralsklerose)
(H) Intrazerebrale Blutung
(I) Läsion des N.hypoglossus
(J) Multiple Sklerose

(K) Hirneigener Tumor
(L) Hirnmetastase
(M) zerebrale Ischämie

7.c In der der von Ihnen notfällig veranlassten zerebralen Computertomographie (CCT) ergibt sich laut neuroradiologischem Befund „Kein Hinweis auf eine frische territoriale Ischämie oder Blutung bei ausgeprägten mikroangiopathischen Veränderungen". Im EKG sehen Sie einen normofrequenten Sinusrhytmus ohne weitere Auffälligkeiten. In der Ultraschalluntersuchung der hirnversorgenden Arterien finden Sie linksseitig eine 80%ige – Stenose der Arteria carotis intera, rechtsseitig eine 60%ige-Stenose. . Der neurologische Befund besteht auch am nächsten Vormittag unverändert weiter, Frau Zimmermann ist weiterhin internistisch beschwerdefrei.
Was ist nun ihre Diagnose? (Long Menu, 1 Antwort aus der folgenden Liste der akzeptierten Möglichkeiten)
Apoplex, Apoplexia, Ischämischer Apoplex, Ischämischer Schlaganfall, Schlaganfall

8 Frau Hofer, eine 28-jährige Patientin in gutem Allgemeinzustand und adipösen Ernährungszustand (1,62m 84kg) wird mit dem Rettungsdienst in die Notaufnahme gebracht. Vor ca. 45 Minuten sei von ihrer Freundin beobachtet worden, wie Frau Hofer plötzlich auf den Boden gefallen sei und dann 1-2 Minuten am ganzen Körper gezuckt habe. Auf Ansprache habe sie danach nicht mehr reagiert. Die Freundin habe dann sofort den Notruf alarmiert. Der Rettungsdienst berichtet dass sie auch eingenässt und sich auf die Zunge gebissen habe. Zurzeit ist die Patientin noch zeitlich und räumlich leicht desorientiert, jedoch wieder kontaktfähig. An das Ereignis und auch an das Eintreffen des Rettungsdienst hat sie keine Erinnerung. Vorerkrankungen sind keine bekannt und sie nimmt außer der Pille keine Medikamente. Sie rauche ca. 5-10 Zigaretten täglich. Ein ähnliches Ereignis gab es bisher noch nicht.
Sie gehen von einem erstmaligen generalisierten Krampfanfall aus. Es finden sich keine Provokations- oder Triggerfaktoren. Frau Hofer gibt jedoch auf Ihre Nachfrage hin progredient zunehmende Kopfschmerzen in den letzten Tagen an, die beim morgendlichen Erwachen am schlimmsten seien.
In der neurologischen Basisuntersuchung (Meningismus, Hirnnerven, Reflexe, Motorik/Kraftgrade, Sensibilität) ergibt sich kein pathologischer Befund.
Auch das Notfall-EKG und das Basis-Notfall-Labor (kleines BB, CRP, Elektrolyte, Kreatinin) sind ohne pathologischen Befund, Frau Hofer ist afebril. Das von den Kollegen der Notaufnahme bereits angemeldete CCT (kraniale Computer-Tomographie) zeigt einen unauffälligen Befund.

8.a Was sind ihre Verdachtsdiagnosen- bzw. was müssen Sie diagnostisch notfallmäßig ausschließen? (Long Menu, 1 Antwort aus der folgenden Liste der akzeptierten Möglichkeiten)
Sinusvenenthrombose, zerebrale Thrombose

8.b Ihre Haupt-Verdachtsdiagnose ist eine Sinusvenenthrombose. Welche Diagnostik ordnen Sie an um Ihren Verdacht zu bestätigen, - bzw. auszuschließen? (Long Menu, 1 Antwort aus der folgenden Liste der akzeptierten Möglichkeiten)
Angiographie Gehirn CT venös, CT-Angiographie Gehirn venös, cMRT mit venöser Gefäßdarstellung, Magnetresonanztomographie-venöse Darstellung der Gefäße, MRT-venöse Darstellung der Gefäße, Magnet-Resonanz-Tomographie-kranial mit venöser

Gefäßdarstellung, CCT mit Venographie, CT-kranial mit Venographie, Computer-Tomographie-cranial mit Venographie, Venöse Darstellung der intrakraniellen Gefäße mittels Schichtbildgebung

8.c Ihre Verdachtsdiagnose Sinusvenenthrombose bestätigt sich in der Bildgebung. Sie antikoagulieren die Patientin daher mit unfraktioniertem Heparin.
Was unternehmen sie therapeutisch bezüglich des epileptischen Anfalls? (Short Menu, 1 Antwort)
(A) Lorazepam i.v. 3x täglich für 3 Monate
(B) Keine antikonvulsive Medikation in den ersten 2 Wochen, nach 2 Wochen dann antikonvulsive Therapie falls weitere Anfälle auftreten
(C) Nur Notfallmedikation mit Diazepam bei Auftreten von weiteren Anfällen.
(D) Bildgebende Kontrolle in 2 Wochen, bis dahin langsame Aufdosierung mit Lamotrigin p.o. beginnen.
(E) Keine Behandlung notwendig, da Sie die Ursache (=Sinusvenenthrombose) bereits behandeln
(F) Nur konservative Behandlung nötig, d.h Meiden von potenziellen Auslösern: Schlafentzug, exzessiver Alkoholgenuss, Drogen, flimmerndes Licht usw.
(G) Anfallsprophylaxe mit Levetiracetam p.o. für 3 Monate, dann Re-evaluation
(H) Kurzzeitige Anfallsprophylaxe mit Phenytoin p.o. bis die Antikoagulation durch Heparin im therapeutischen Bereich ist

9 Herr Bismarck, ein 52-jähriger Patient in gutem Allgemeinzustand und gutem Ernährungszustand (1,74m; 75kg) stellt sich in der Notfall-Ambulanz wegen seit 10 Tagen bestehender, hefiger Rückenschmerzen und seit gestern zusätzlich bestehender „Fußschwäche“ vor. Auf der Überweisung des Hausarztes steht: „V.a. L4-Syndrom“, das mitgebrachte MRT der LWS von heute Morgen ist jedoch ohne pathologischen Befund. In der klinischen Untersuchung finden Sie rechts eine Fußheber- und Fußsenker-parese (beides Kraftgrad 4/5) mit fraglichem Sensibilitätsdefizit L5/S1, sowie einen abgeschwächten Achillessehnenreflex rechts. Das Lasegúe-Zeichen ist beidseits negativ. Der restliche neurologische Befund in der Basisuntersuchung (Hirnnerven, Meningismus, Motorik, Muskeleigenteflexe, Sensorik, Koordination) ist unauffällig.

9.a Welche anamnestischen Angaben von Herrn Bismarck helfen Ihnen für die weitere Diagnose-Findung am besten? (Short Menu, 2 Antworten)
(A) Meningitis als Kind gehabt
(B) Neu aufgetretene Antriebsarmut
(C) Hatte eine Magen-Teil-Resektion vor 10 Jahren
(D) Eine Tante hatte ALS (Amyotrophe Lateralsklerose)
(E) Die Schmerzen sind v.a. nachts
(F) Lesen strengt ihn mehr an als früher
(G) Geht einer schweren körperlichen Arbeit nach
(H) Ernährt sich seit 2 Jahren vegan
(I) Stent in der linken Arteria femoralis bei pAVK (periphere arterielle Verschlusskrankheit) seit 2 Jahren
(J) Arterieller Hypertonus, eingestellt mit 2-facher antihypertensiver Medikation
(K) Macht regelmäßige Wanderungen im Schwarzwald

9.b Herr Bismarck gibt an, dass die Schmerzen v.a. nachts seien. In der notfällig durchgeführten Laboruntersuchung mit kleinem Blutbild, Gerinnungswerten Nieren-/Leber-/Schilddrüsenwerten und C-reaktivem Protein ergeben sich durchwegs Normalbefunde, in der elektrophysiologischen Zusatzdiagnostik (EMG und sensible Neurographien) ergeben sich ebenfalls ausschließlich Normalbefunde. Sie vermuten eine Radikulitis.
Welche weitere Diagnostik ist nun indiziert? (Long Menu, 1 Antwort aus der folgenden Liste der akzeptierten Möglichkeiten)
Liquorpunktion, LP, Lumbalpunktion

9.c In der notfälligen Liquordiagnostik finden Sie eine lymphozytäre Pleozytose (230/µl; Norm: <5 Zellen/µl) und ein erhöhtes Gesamtprotein (1200mg/l Norm: < 400mg/l).
Was sind Ihre 2 Haupt-Verdachtsdiagnosen? (Long Menu, 2 Antworten aus der folgenden Liste der akzeptierten Möglichkeiten)
Borreliose, Varizella-zoster-Virus, VZV, Borrelien, Borrelia burgdorferi, Neuroborreliose, Herpes zoster

9.d Sie finden in der ausführlichen Liquordiagnostik eine intrathekale 3-Klassen-Synthese. Die VZV (Varizella-Zoster-Virus)-PCR (Polymerase-Kettenreaktion) ist negativ, es finden sich in der zytopathologischen Untersuchung keine pathologischen Zellen. Sie haben daher den Verdacht einer Neuroborreliose.
Was sind die zwei adäquaten Therapieoptionen? (Short Menu, 2 Antworten)
(A) Ciprofloxacin i.v. für 6 Monate
(B) Ceftriaxon p.o. für 6 Monate
(C) Dexamethason i.v. für 14 Tage
(D) Ceftriaxon i.v. für 14 Tage
(E) Ciprofloxacin p.o. für 14 Tage
(F) Keine Therapie notwendig, da die Krankheit selbstlimitierend ist
(G) Keine Therapie möglich, da die Infektion schon zu lange zurück liegt
(H) Nur symptomatische Therapie notwendig
(I) Doxycyclin 3x 100mg p.o für 21 Tage
(J) Chinin + Clindamycin p.o.
(K) Diclofenac p.o. für 14 Tage

10 Frau Kirchhofer, eine 21-jährige Jura-Studentin in gutem Allgemeinzustand und schlankem Ernährungszustand (1,74m; 60kg) stellt sich bei Ihnen in der neurologischen Praxis vor. Seit 4 Monaten (ungefähr seit sie ihr Studium begonnen hat und von zu Hause ausgezogen sei) bestehen immer wiederkehrende Drehschwindelattacken, ca. drei Mal im Monat. Sie bekomme dann auch immer starke, pulsierende Kopfschmerzen, wenn sie versuchen würde weiter zu lernen – was sie auch wiederholt versucht hätte, da bald die Klausuren anstehen würden und sie jede freie Minute brauche. Wenn sie sich dann aber doch hinlegen würde, würden der Schwindel und die Kopfschmerzen nach 1-2 Stunden verschwinden. Einen Hörverlust während der Attacken verneint sie. Sie sagt, sie sei jetzt zu Ihnen gekommen, weil sie das dringend in den Griff bekommen müsse vor ihren Klausuren. Vorerkrankungen sind keine bekannt, sie nimmt auch keine Medikamente und hatte noch keine Operationen. Im von der Patientin mitgebrachten MRT (Magnetresonanz-Tomographie) wie auch in der bereits ambulant durchgeführten HNO-ärztlichen Abklärung finden sich keine
Auffälligkeiten.

Ihre neurologische Untersuchung incl. Nystagmusprüfung mit Frenzelbrille, Kopfimpuls-Test und Testung auf eine Skew deviation sind sämtlich unauffällig.

10.a Was sind Ihre 2 wahrscheinlichsten Arbeitsdiagnosen? (Long Menu, 2 Antworten aus der folgenden Liste der akzeptierten Möglichkeiten)
Migräne, Psychische Faktoren, Psychogen, Psychosomatisch, Somatisierungsstörung, Vestibuläre Migräne, Psychogener Schwindel

10.b Welcher der folgenden Angaben müssen Sie aufgrund Ihrer Verdachtsdiagnosen und der Anamnese die größte Bedeutung beimessen? (Short Menu, 1 Antwort)
(A) Allergie gegen Gräser und Pollen
(B) Trinkt am Wochenende meist 1-2 Bier
(C) Hat vom 16-18. Lebensjahr gelegentlich Cannabis konsumiert
(D) Raucht 1/2 Schachtel Zigaretten am Tag
(E) War vor drei Monaten in Indien
(F) Hat einen Vogel als Haustier
(G) Hat die letzten Wochen diffuse Gelenkschmerzen
(H) Hat seit 3 Tagen leichte Diarrhoe
(I) Positive Familienanamnese bezüglich Brust-Krebserkrankungen (Oma)
(J) Positive Familienanamnese bezüglich Herzinfarkte (Opa)
(K) Positive Familienanamnese bezüglich Schizophrenie (Onkel)
(L) Positive Familienanamnese bezüglich Migräne (Mutter)
(M) Gewichtsverlust von 1-2kg im letzten halben Jahr
(N) fühlt sich in den letzten 2 Monaten abgeschlagen und energieärmer
(O) Temperatur von 37,6°C

10.c Die Patientin berichtet, dass ihre Mutter im Alter von 20 bis 40 Jahren stark unter Migräne gelitten hätte. Sie stellen die Verdachtsdiagnose einer vestibulären Migräne bei Frau Kirchhofer.
Was geben Sie der Patientin als Akut-Therapie, sollte erneut eine Attacke auftreten? (Short Menu, 2 Antworten)
(A) Acetylsalicylsäure 1000mg
(B) Acetylsalicylsäure 100mg
(C) Valproat
(D) ß-Blocker
(E) Magnesium
(F) Triptane
(G) Paracetamol 125mg
(H) Dexamethason 1000mg

10.d Sie haben der Patientin ASS 1000mg sowie Triptane bei Bedarf verordnet und sie gebeten ein Migräne-Tagebuch zu führen und sich mit diesem in 2 Monaten erneut vorzustellen. Bei diesem Termin sehen Sie Frau Kirchhofer nun erneut. Sie berichtet, dass ihr die Medikamente sehr gut geholfen haben, sie jedoch erneut drei bzw. fünf Attacken pro Monat hatte. Sie wollen nun eine zusätzliche medikamentöse Prophylaxe einleiten.
Welche kommen hier in Frage. Markieren Sie 2!
(Short Menu, 2 Antworten)
(A) Propanolol

(B) Lidocain
(C) Phenytoin
(D) Dihydroergotamin
(E) Metoprolol
(F) Prednison
(G) Amitriptylin
(H) ACE-Hemmer
(I) Pregabalin
(J) Clobazam
(K) Citalopram
(L) Acetylsalicylsäure

11 Herr Best, ein 33-jähriger Patient in reduziertem Allgemeinzustand und magerem Ernährungszustand (1,85m; 67kg) wird von einem Freund in die Notaufnahme gebracht. Der Freund mache sich sorgen, da Herr Best seit heute morgen verändert wäre und manchmal "verwirrt". Herr Best selbst sagt er "fühle sich nicht wohl". Er ist wach, orientiert zu Person und Ort, jedoch unsicher zur Situation ("Arztpraxis") und Zeit ("10.01. statt 17.01."). Herr Best wirkt sehr nervös und unruhig. Sie bemerken ein feinschlägiges Zittern, die Hände sind stark verschwitzt. Der Blutdruck ist mit 180/100mmHg deutlich erhöht, am Monitor zeigt sich eine Sinustachykardie von 112/min. Der Blutzucker beträgt 98 mg/dl. Herr Best ist afebril. Der restliche internistische Befund ist regelgerecht. In der neurologischen Untersuchung ergeben sich keine Hinweise auf fokale Defizite. Herr Best verneint Vorerkrankungen oder Operationen, er nimmt keine regelmäßigen Medikamente ein.
In der Akte der Notaufnahme finden Sie 4 Aufenthalte wegen Alkohol-Intoxikationen in den letzten 6 Monaten. Auf Ihre Fragen hin versichert er Ihnen, in den letzten 2 Tagen keine Drogen oder Alkohol zu sich genommen zu haben. Dazu passend ist der Blutalkoholspiegel im Notfall-Labor 0,0g/l. Die restlichen Labor-Werte sind bis auf leicht erhöhte Leberwerte komplett unauffällig.

11.a Nennen Sie die 2 wahrscheinlichsten Verdachtsdiagnosen! (Long Menu, 1 Antwort aus der folgenden Liste der akzeptierten Möglichkeiten)
Alkoholentzugs-Syndrom, Entzugssyndrom

11.b In der weiteren ausführlichen Anamnese berichtet Herr Best auf die Frage nach seinem Alkoholkonsum dass er seit vorgestern „trocken“ sei (sonst laut eigenen Angaben seit ca. 2 Jahren 8-10 Bier am Tag) und „jetzt endlich mit dem Alkohol aufhören möchte.“ Er verneint erneut die Einnahme sonstiger Drogen. Sie vermuten ein Alkohol-Entzugssyndrom. Sie wollen Herrn Best zur weiteren Behandlung stationär aufnehmen. Was sind die in diesem Fall notwendigen medikamentösen Maßnahmen? Nennen Sie 2 Medikamente aus unterschiedlichen Substanzklassen. (Long Menu, 2 Antworten aus der folgenden Liste der akzeptierten Möglichkeiten)
Clomethiazol (z.B. Distraneurin®) (Sedativa), Diazepam (z.B. Valium®, Valiquid®) (Benzodiazepine), Lorazepam (z.B. Tavor®) (Benzodiazepine), Vitamin B1 (z.B. Betabion®)

11.c Herr Best verlässt kurze Zeit später die Notaufnahme gegen ärztlichen Rat. Zehn Stunden später wird er von seinem Freund in deutlich verschlechtertem Zustand wieder gebracht. Herr Best ist agitiert und desorientiert, kann jedoch noch mit Ihnen kommunizieren. Er berichtet "weiße Mäuse zu sehen." Sie stellen einen Blutdruck von 200/120 mmHg und einen Puls von 125/min fest.

Welche der folgenden therapeutischen Optionen ist in diesem Fall die Beste? (Short Menu, 1 Antwort)
(A) Aufnahme Intensivstation, Vitamin B12, Clomethiazol, Haloperidol und Carbamazepin
(B) Aufnahme Intensivstation, Vitamin B1, Clomethiazol und Carbamazepin
(C) Aufnahme Intensivstation, Vitamin B12, Haloperidol und Carbamazepin
(D) Aufnahme Intensivstation, Vitamin B1, Levetirazetam und Clonidin
(E) Aufnahme Intensivstation, Vitamin B12, Clonidin und Haloperidol
(F) Aufnahme Intensivstation, Vitamin B1, Clomethiazol, Haloperidol und Clonidin
(G) Entlassung nach Hause, Vitamin B12, Clomethiazol, Haloperidol und Carbamazepin
(H) Entlassung nach Hause, Vitamin B1, Clomethiazol und Carbamazepin
(I) Entlassung nach Hause, Vitamin B12, Haloperidol und Carbamazepin
(J) Entlassung nach Hause, Vitamin B1, Levetirazetam und Clonidin
(K) Entlassung nach Hause, Vitamin B12, Clonidin und Haloperidol
(L) Entlassung nach Hause, Vitamin B1, Clomethiazol und Haloperidol
(M) Aufnahme Normalstation, Vitamin B12, Clomethiazol, Haloperidol und Carbamazepin
(N) Aufnahme Normalstation, Vitamin B1, Clomethiazol und Carbamazepin
(O) Aufnahme Normalstation, Vitamin B12, Haloperidol und Carbamazepin
(P) Aufnahme Normalstation, Vitamin B1, Levetirazetam und Clonidin
(Q) Aufnahme Normalstation, Vitamin B12, Clonidin und Haloperidol
(R) Aufnahme Normalstation, Vitamin B1, Clomethiazol und Haloperidol

12 Karl Marwick, ein 20jähriger Patient, wird vom Rettungsdienst in die Notaufnahme gebracht. Seine Freundin habe beobachtet, wie Herr Marwick auf der Couch liegend "um sich geschlagen habe",- sowie Schaum vor dem Mund gehabt habe. Daraufhin habe sie den Rettungsdienst alarmiert, der Ihnen jetzt Herr Marwick bringt. Herr Marwick ist bei Eintreffen wach, hat jedoch keine Erinnerung für das Ereignis. Er hatte keine ähnlichen Episoden in der Vorgeschichte. Sie fragen die Freundin genauer nach dem Akut-Ereignis.

12.a Welche der folgenden Antwortmöglichkeiten würden am ehesten für einen epileptischen Anfall sprechen? (Short Menu, 3 Antworten)
(A) Augen offen, starr blickend
(B) Dauer 5-10 Minuten
(C) schlaffer Körpertonus
(D) Augen zugekniffen
(E) Dauer >10 Minuten
(F) Dauer 1-2 Minuten
(G) Augen offen, nach oben verdreht
(H) Lateraler Zungenbiss
(I) Reorientierung innerhalb weniger Sekunden

12.b Die neurologische Basisuntersuchung (Meningismus, Hirnnerven, Motorik, Sensibilität und Reflexe) ist ohne pathologischen Befund. Was veranlassen Sie (notfallmäßig) als erste (Zusatz-) Diagnostik? (Long Menu, 1 Antwort aus der folgenden Liste der akzeptierten Möglichkeiten)
Labor, Blutabnahme

12.c Das Notfall-Labor von Herrn Marwick ist völlig unauffällig. Herr Marwick berichtet auf Ihre Nachfrage, vor dem Anfall keinen Infekt mit Fieber oder starke Kopfschmerzen bemerkt

zu haben, seine Freundin bestätigt, dass er „so wie immer“ gewesen wäre. Sie explorieren mögliche Provokationsfaktoren.
Welcher der folgenden Punkte könnte den epileptischen Anfall provoziert haben? (Short Menu, 1 Antwort)
(A) In den letzten 24 Stunden relativ wenig Flüssigkeit (ca. 1,5l) zu sich genommen
(B) In den letzten 24 Stunden nur ein Frühstück und ein Stück Pizza zu sich genommen
(C) Aufgrund des Beginn der Fastenzeit seit 10 Tagen kein Alkohol mehr getrunken (sonst 1 Flasche Bier (0,33l) am Tag in den letzten 6 Monaten)
(D) Ausgeprägter Zigaretten-Konsum (>30 Zigaretten/ Tag)
(E) Bis vor 2 Jahren regelmäßiger Cannabis-Konsum (1-2 Joints alle 2 Wochen).
(F) Einmalig Amphetamin-Konsum (Speed) vor ca. 6 Monaten.
(G) Einnahme eines Opioids (Tilidin + Naloxon) aufgrund von lumbalen Rückenschmerzen seit 2 Wochen.
(H) Hat heute Nacht nur 6 Stunden geschlafen (sonst durchschnittlich 7-8 Stunden pro Nacht)
(I) Ungewohnt starke sportliche Betätigung (2 Stunden Fußballspielen) am Vortag.
(J) Keiner der genannten Punkte

12.d Welche der folgenden Vorgehensschritte bzw. Empfehlungen bezüglich Diagnostik, Therapie und Fahreignung sind aufgrund Herrn Marwicks Angaben notwendig? (Short Menu, 3 Antworten)
(A) Entlassung ohne weitere Abklärung, da es sich um einen Gelegenheitsanfall handelt.
(B) Entlassung nach Hause mit Empfehlung der ambulanten Abklärung (EEG, cMRT)
(C) Notfällige Aufnahme auf die Intensivstation zur Überwachung
(D) Notfällige Durchführung einer CCT (kraniale Computer-Tomographie) mit Kontrastmittel in der Notaufnahme.
(E) Notfällige Lumbalpunktion in der Notaufnahme.
(F) Keine spezifische Therapie, aber Verbot von Ausdauersport
(G) Vorerst keine antikonvulsive Medikation, nur Aufklärung über Vermeidung von Provokations- und Triggerfaktoren
(H) Sofortiger Beginn einer Dauer-Therapie mit Valproat
(I) Sofortige hochdosierte Gabe von Lorazepam i.v. auf der Intensivstation
(J) Sofortiger Beginn einer langsamen Eindosierung von Lamotrigen
(K) Aufklärung über Fahruntauglichkeit für mindestens 12 Monate
(L) Fahrverbot bis die erforderlichen Antikonvulsiva-Spiegel erreicht sind
(M) Entzug des Führerscheins aufgrund der Diagnose: Epilepsie
(N) Aufklärung über Fahruntauglichkeit für mindestens 6 Monate
